# Microtubules in *Bacteria*: Ancient Tubulins Build a Five-Protofilament Homolog of the Eukaryotic Cytoskeleton

**DOI:** 10.1371/journal.pbio.1001213

**Published:** 2011-12-06

**Authors:** Martin Pilhofer, Mark S. Ladinsky, Alasdair W. McDowall, Giulio Petroni, Grant J. Jensen

**Affiliations:** 1 California Institute of Technology, Pasadena, California, United States of America; 2 Howard Hughes Medical Institute, Division of Biology, Pasadena, California, United States of America; 3 Dipartimento di Biologia, University of Pisa, Pisa, Italy; MRC Laboratory of Molecular Biology, United Kingdom

## Abstract

Microtubules play crucial roles in cytokinesis, transport, and motility, and are therefore superb targets for anti-cancer drugs. All tubulins evolved from a common ancestor they share with the distantly related bacterial cell division protein FtsZ, but while eukaryotic tubulins evolved into highly conserved microtubule-forming heterodimers, bacterial FtsZ presumably continued to function as single homopolymeric protofilaments as it does today. Microtubules have not previously been found in bacteria, and we lack insight into their evolution from the tubulin/FtsZ ancestor. Using electron cryomicroscopy, here we show that the tubulin homologs BtubA and BtubB form microtubules in bacteria and suggest these be referred to as “bacterial microtubules” (bMTs). bMTs share important features with their eukaryotic counterparts, such as straight protofilaments and similar protofilament interactions. bMTs are composed of only five protofilaments, however, instead of the 13 typical in eukaryotes. These and other results suggest that rather than being derived from modern eukaryotic tubulin, BtubA and BtubB arose from early tubulin intermediates that formed small microtubules. Since we show that bacterial microtubules can be produced in abundance in vitro without chaperones, they should be useful tools for tubulin research and drug screening.

## Abstract 2

The unequivocal identification of microtubules in bacteria throws light on the evolution of modern eukaryotic microtubules from a primordial structure.

## Author Summary

Bacteria are generally distinguished from the cells of fungi, plants, and animals (eukaryotes) not only by their much smaller size but also by the absence of certain subcellular structures such as nuclei, internal organelles, and microtubules. Using state-of-the-art microscopy, we demonstrate here that microtubules do exist in some bacteria. These bacterial microtubules are built from proteins that are closely related to the microtubule proteins in eukaryotes. Bacterial microtubules are smaller in diameter than their counterparts in eukaryotic cells but have the same basic architecture. We propose that bacterial microtubules represent primordial structures that preceded eukaryotic microtubules evolutionarily. Because bacterial microtubules can be produced and handled in the lab more easily than their eukaryotic counterparts, they may become useful tools for microtubule research and anti-cancer drug screening.

## Introduction

Microtubules are among the most-studied eukaryotic subcellular structures [1]–[4]. Their crucial role in cell division, transport, and motility make them superb targets for anti-cancer drugs. All tubulins evolved from a common ancestor they share with the distantly related bacterial cell division protein FtsZ [5]–[9], but while eukaryotic α- and β-tubulins evolved into highly conserved tube-forming heterodimers [1],[4], bacterial FtsZ presumably continued to function as single homopolymeric protofilaments as it does today [10]. Although unidentified tubular structures have been seen in certain bacteria [3], tubulin genes have not been found in the genomes. The discovery of bacterial tubulin A (BtubA) and bacterial tubulin B (BtubB) in several *Prosthecobacter* strains was therefore exciting, since BtubA and BtubB are much more closely related to eukaryotic tubulins than to any other bacterial protein [11],[12]. Prosthecobacters belong to the *Planctomycetes*-*Verrucomicrobia*-*Chlamydiae* superphylum, whose members have been shown to possess various eukaryote-like features [13]–[15]. The function of BtubA/B in *Prosthecobacter* remains unclear, however, since they coexist with genuine FtsZ and are therefore unlikely to be the major cell division proteins [12],[16].

Since genomic organization and other evidence suggest prosthecobacters most probably acquired the *btubAB* genes by horizontal gene transfer [11],[12],[16]–[19], BtubA/B have been suggested to be descendants of modern eukaryotic α- and/or β-tubulins [6],[11],[17],[19],[20]. More recently, however, it was argued that they represent an ancient form, since (i) like FtsZ, BtubA/B assembles in diverse conditions and (ii) both BtubA and BtubB contain α- *and* β-tubulin-like features [21]. Just like α- and β-tubulins, BtubA/B form heterodimers which polymerize into protofilaments in vitro. Typically, 13 α/β-protofilaments align slightly staggered to form a hollow eukaryotic microtubule, but microtubule-like structures have not been described in BtubA/B preparations [17],[19],[21]. Cytoskeletal structures were also not observed in *Prosthecobacter dejongeii* cells by conventional thin-section electron microscopy (EM) [11],[22].

Reasoning that the structure of BtubA/B filaments might not have been preserved in vivo by conventional EM methods, here we sought to characterize BtubA/B structures using electron cryotomography (ECT) [23]. We show that BtubA/B form five-protofilament microtubules in vivo. Together with additional phylogenetic sequence analyses, these results support the notion that BtubA/B microtubules represent an ancient evolutionary form that led to modern eukaryotic 13-protofilament microtubules.

## Results and Discussion

*btubA* and *B* genes are found in certain *Prosthecobacter* species including *P. vanneervenii*, *P. dejongeii*, and *P. debontii*, but not *P. fluviatilis* [11],[12],[24]. To begin, we verified that BtubA and BtubB proteins are in fact expressed in the species where the genes are present (Figures S1 and S2). Western hybridization and PCR also confirmed the absence of BtubA and BtubB in *P. fluviatilis* (Figure S2) [24].

**Figure 1:**
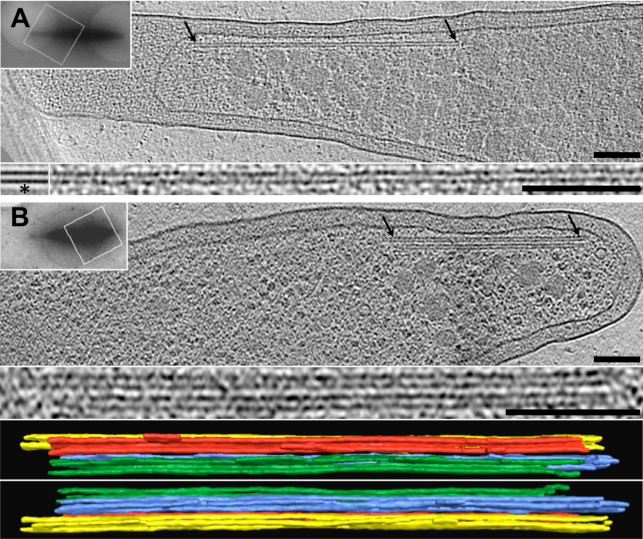
Cytoskeletal BtubA/B-candidate structures imaged in *Prosthecobacter*. *Prosthecobacter vanneervenii* cells showing tube-like BtubA/B-candidate structures occurring (A) individually or (B) in a bundle. Shown are 11-nm thick slices through cryotomograms. Arrows indicate cytoskeletal structures, which are also shown enlarged below. Asterisk in panel A identifies a sub-tomographic average. Upper-left insets show low-magnification overviews of the cells; rectangles indicate areas imaged in 3-D. Bottom: 3-D segmentation of the bundle of panel B shown from two views (four tubes are present). Scale bars are 100 nm. See Figure S3 for further examples of BtubA/B structures.

Next, *Prosthecobacter* cells were grown under different conditions and plunge-frozen across EM grids. A total of 589 cells were then imaged in 3-D by ECT. The spindle-shaped cells were polymorphic and exhibited prosthecae (cellular stalks) of different lengths. As seen in other bacterial phyla [25], multiple classes of cytoskeletal structures were seen, but one class had a tube-like morphology and was frequently found in the harboring species, but never in the *btubAB*-lacking strain (Figure 1). The abundance of these tube-like structures was dependent on the species imaged as well as the growth conditions and growth stage, and was found to be highest in *P. vanneervenii* cells grown directly on EM grids (67% of cells imaged). In sum, the tube-like structures were found in 48 of 176 *P. vanneervenii*, 9 of 111 *P. dejongeii*, 15 of 151 *P. debontii*, and 0 of 151 *P. fluviatilis* cells. The tube-like structures were 200–1,200 nm long, always parallel to the cytoplasmic membrane, almost always localized in the stalk or in the transition zone between stalk and cell body, and occurred either individually or in bundles of two, three, or four (Figure 1, Figure S3, Movie S1). Chemical fixatives were found to degrade the structures (Figure S4), explaining why they were likely missed in previous conventional EM studies [11],[22].

**Figure 2:**
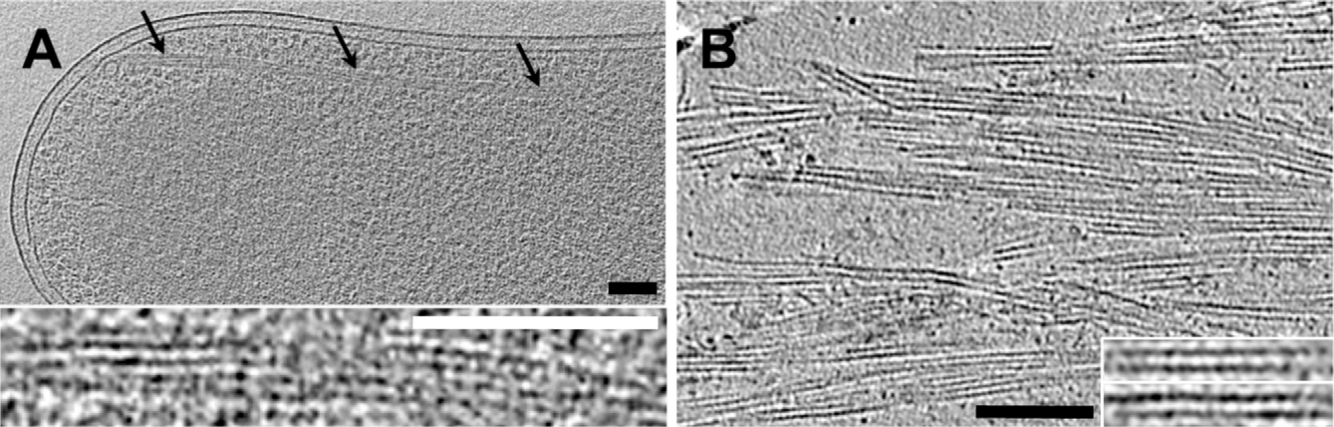
Recombinant BtubA/B structures resemble the tube-like structures imaged in *Prosthecobacter*. (A) *E. coli* cell co-expressing BtubA and BtubB (from *P. dejongeii*) and (B) recombinant BtubA/B polymerized in vitro exhibiting tube-like densities which are strikingly similar to those seen in *Prosthecobacter*. Shown are 11-nm thick slices through electron cryotomograms. Arrows indicate cytoskeletal structures. Black scale bars and white scale bar (applies to enlarged images) are 100 nm.

Since genetic tools are not yet available for prosthecobacters, we applied labeling and heterologous expression approaches to test whether the candidate structures were in fact composed of BtubA/B as expected by their correlation with the presence of the genes. Recombinant *Escherichia coli* cells co-expressing BtubA and BtubB were imaged by ECT and exhibited strikingly similar tube-like structures running the length of the cells (Figure 2A) with the same localization as had been reported for BtubA/B from immuno-fluorescence [19]. Tube-like structures were not seen in control *E. coli* cells not expressing ButbA/B. Nearly identical tube-like structures were also seen when recombinant BtubA/B was polymerized in vitro and imaged by ECT (Figure 2B). The diameters and subunit repeat distances of all three structures (in *Prosthecobacter*, recombinant *E. coli*, and in vitro) were similar (7.6, 7.7, and 7.6 nm diameters, and 4.4, 4.4, and 4.2 nm repeat distances, respectively) (Figures 1, 2, and S3). Finally, immunogold-staining using anti-BtubB antibodies localized the proteins to the same region of *Prosthecobacter* cells as the candidate structures seen by ECT (Figures S5 and S6). We conclude therefore that the tube-like structures are composed of BtubA/B, and the slight differences in repeat distance, straightness, and bundling in the three samples were due to differences in protein concentrations and/or the absence of other interacting proteins in vitro and in *E. coli*.

**Figure 3:**
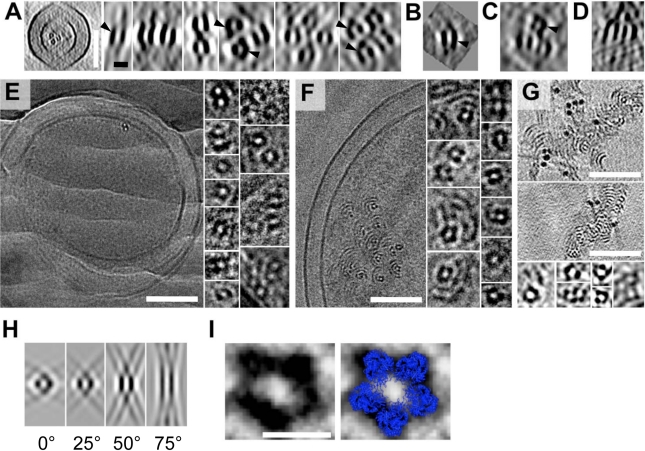
BtubA/B assembles into five-protofilament tubes. (A–D) Tomographic slices showing cross-sectional views of BtubA/B tubes in (A) prosthecobacters, (B) a sub-tomographic average from *P. vanneervenii*, (C) *E. coli* co-expressing BtubA/B (from *P. vanneervenii*), and (D) BtubA/B polymerized in vitro. (E, F) Images and (G) tomographic slices through cryosectioned, high-pressure-frozen (E) *P. vanneervenii* cells, (F) *E. coli* cells co-expressing BtubA/B, and (G) BtubA/B polymerized in vitro, showing that the BtubA/B structures are complete tubes. (H) Slices through simulated tomograms showing cross-sectional views of five-protofilament tube models lying in a plane perpendicular to the electron beam at different angles to the tilt-axis (from left to right 0°, 25°, 50°, 75°), showing how the well-known missing wedge effect recapitulates the apparent lack of density in the tops and bottoms of the tubes seen in the tomograms. (I) Pseudo-atomic model of a five-protofilament bacterial microtubule (blue; built from Protein Data Bank structure 2 btq) superimposed on the image of a cryo-sectioned BtubA/B tube (left). The tomographic slices are (A, C) 114 nm, (B, H) 11 nm, (D) 76 nm, and (G) 88 nm thick. The black scale bar is 10 nm and applies to enlarged images and simulations in panels A–H; white scale bars are 100 nm in panels E–G and 10 nm in panel I.

We have described the BtubA/B structures so far as “tube-like” because when acquiring a cryo-tomographic tilt-series, images of samples tilted beyond ∼65° cannot generally be included, so there is a missing “wedge” of data in reciprocal space that reduces the resolution in the direction of the electron beam. As a result, the “top” and “bottom” boundaries of cylindrical objects (considering the electron beam to be “vertical”) are smeared, leaving the sidewalls to appear like two arcs facing each other (Figure 3A–D). Because the opposing arcs observed here were *always* in this orientation (facing each other and the beam path), it was clear that the structures must have been complete tubes distorted by the missing wedge rather than, for instance, parallel protofilaments, which would not be expected to always orient themselves in the same direction with respect to the electron beam. Nevertheless different orientations of tubes with respect to the tilt axis aggravate the missing wedge artifact differently [26],[27], so to explore this effect tomograms of a known, tubular input structure consisting of BtubA/B crystal structures (see below) were simulated at different angles with respect to the tilt axis. These simulations recapitulated the experimental results well, since the density patterns (Figure 3H) were highly similar to those seen in experimental tomograms.

To further confirm that the BtubA/B structures were in fact complete tubes and to obtain clearer cross-sectional views, *btubAB*-harboring *Prosthecobacter* cells, recombinant *E. coli* cells, and purified BtubA/B polymerized in vitro were all high-pressure-frozen, cryosectioned, and imaged (Figure 3E–G). Cryosections through BtubA/B tubes appeared pentagonal, suggesting five-protofilament tubes. Using the heterodimeric BtubA/B crystal structure [17], we produced tube models with four, five, and six protofilaments for comparison. To maintain reasonable lateral interactions in such small tubes, protofilaments had to be spaced slightly closer (4.6 nm) than protofilaments in eukaryotic microtubules (5 nm), and this resulted in tube diameters of 6.7, 7.8, and 9.2 nm, respectively, for four-, five-, and six-protofilament tubes. Thus only the five-protofilament model was consistent with the 7.6-nm diameter measured in the tomograms, and the five-protofilament model fit the density of the BtubA/B tubes compellingly well (Figure 3I). Cross-sectional views of BtubA/B tubes in cryo-tomograms of whole cells and sub-tomogram averages often showed a left-right asymmetry (arrowheads in Figure 3A–C). Such an asymmetry can only arise from an uneven number of protofilaments, as demonstrated by simulated tomograms (Figure S7), further suggesting five rather than four or six protofilaments. Because the left-right asymmetries in computational projections and in sub-tomographic averages at different positions along the tube axis remained consistent, the five protofilaments must be straight rather than twisting around the tube (Figure S8).

Previous EM images of negatively stained, recombinant BtubA/B polymerized in vitro were not described as tubes, but as protofilament bundles or twisted pairs [17],[19],[21]. We obtained similar-looking images staining our own purified BtubA/B (Figure S9), but having observed clear tubes in vivo and noting the frequent pairing of parallel densities ∼7.6 nm apart in both our negatively stained images and the previously published images, we believe all these samples contained five-protofilament tubes as well. The alternative (two protofilaments 7.6 nm apart) seems unlikely since BtubA/B protofilaments are known to be only 4 nm in diameter [17], and would therefore have to be closer together to interact. Slight helical twists in the tubes in vitro may have caused the appearance of twisted pairs [17].

**Figure 4:**
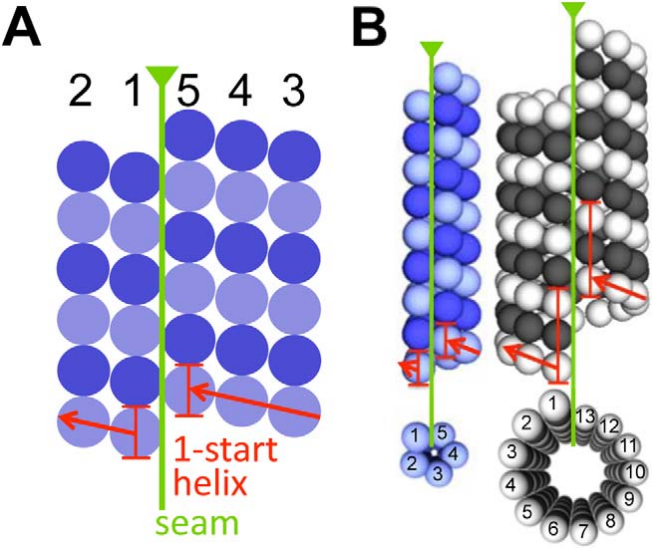
Structural model of “bacterial microtubules.” (A) 2-D schematic of the proposed architecture of bacterial microtubules built from BtubA (dark-blue) and BtubB (light-blue). Protofilaments are numbered 1–5. (B) 3-D comparison of the architectures of a bacterial microtubule (left; BtubA in dark-blue; BtubB in light-blue) and a 13-protofilament eukaryotic microtubule (right; β-tubulin in black; α-tubulin in white). Seams and start-helices are indicated as in (A).

**Figure 5:**
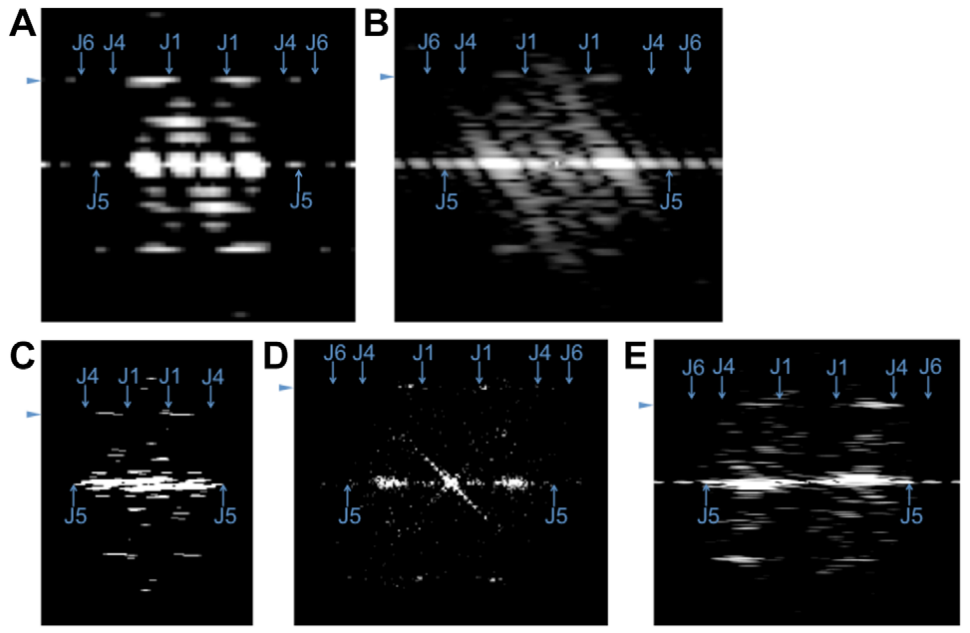
BtubA/B tubes have a helical, microtubule-like lattice. (A) Fourier transform of a simulated projection image (1.2 nm/pixel) of a five-protofilament BtubA/B-tube model (Figure 4) with a helical, microtubule-like lattice. A prominent pair of elongated spots on the subunit-repeat layer line on either side of the meridian corresponds to the helical family J1. Pairs of spots for the helical families J4 and J6 were very weak, likely because of destructive interference with the first minimum of the J1 Bessel-function. The subunit-repeat layer line was surprisingly asymmetric probably because of the small number of protofilaments and the resulting lack of an extended “front” and ”back” side. The asymmetry also shifted around the meridian depending on the rotation of the tube around its length axis (Figure S10). (B–E) Fourier transforms of BtubA/B-tubes in (B) a 2-D slice through a subtomogram average (from within a *P. vanneervenii* cell), (C) a negatively stained projection image (of an in vitro assembled tube), (D) a cryo-EM projection image (of an in vitro assembled tube), and (E) a 2-D tomographic slice containing an in vitro assembled tube. The prominent pair of J1 spots on the subunit repeat layer line in all cases suggests a helical lattice, as all non-helical models lead to high-intensity spots *on* the meridian (unpublished data). Arrowheads indicate the subunit repeat layer line. Arrows mark the maxima of the J1, J4, J5, and J6 Bessel-functions, assuming outer rather than mass-weighted radii (and therefore marking the expected meridional borders of spots).

While the number of protofilaments in eukaryotic microtubules can vary, the lateral interactions between them are conserved [28] such that each protofilament is shifted 0.93 nm along the tube axis relative to its neighbors. In 13-protofilament microtubules, this shift results in a three-start helix around the microtubule and a seam where α- and β-subunits interact [29]. Because the loops that are involved in these interactions are also present in BtubA and BtubB [17], we expect BtubA/B protofilaments to be shifted similarly. The sum of five such shifts (4.65 nm) is similar to the subunit repeat distance measured in BtubA/B tubes (4.2 and 4.4 nm, respectively) and suggests that BtubA/B form one-start helical tubes (Figure 4). The difference could be accommodated by a slightly different lateral interaction (a stagger of 0.84–0.88 nm instead of 0.93 nm). In support of this model, the major features of Fourier transforms of BtubA/B tube images matched those of a one-start five-protofilament helix model (Figures 5 and S10), but did not clarify whether BtubA/B tubes have an “A-lattice” without seam or a “B-lattice” with seam [30]. The latter seems more likely, however, since the B-lattice has been resolved in eukaryotic 13-protofilament microtubules, and is therefore depicted in Figure 4. Based on our data, the BtubA/B crystal structure [17], and the known structural features of the eukaryotic microtubule, we conclude therefore that BtubA/B heterodimers form five-protofilament, one-start helical tubes in vivo with lateral and longitudinal interactions like their eukaryotic counterparts. Since BtubA/B are true homologs of eukaryotic tubulin [11],[12],[17] and they form closely related structures differing mainly in the number of protofilaments, we suggest they be referred to as “bacterial microtubules” (bMTs).

**Figure 6:**
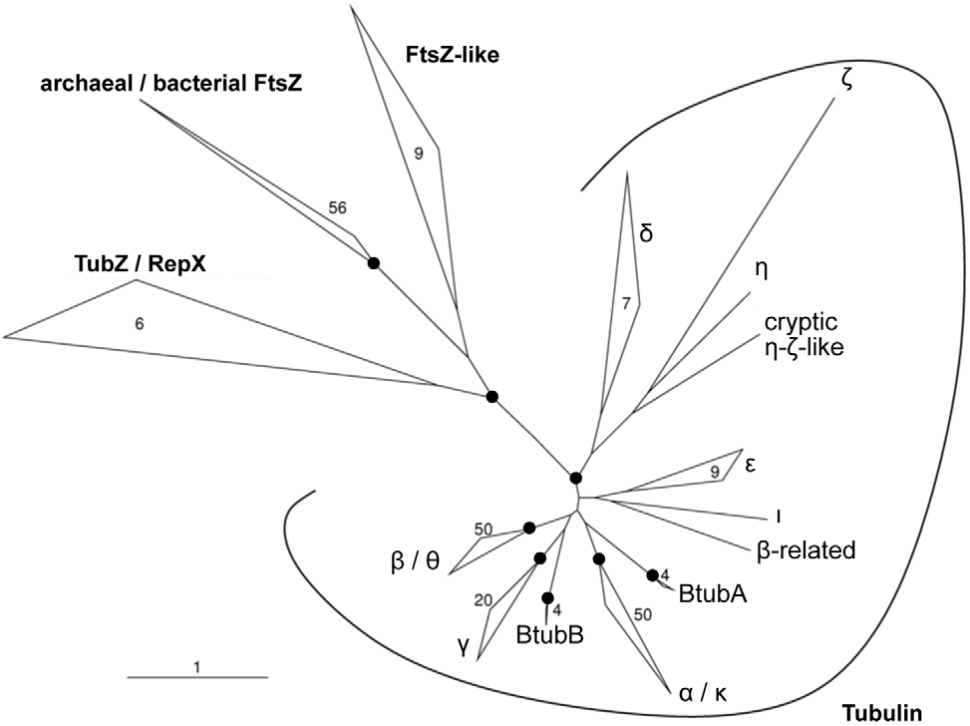
BtubA and BtubB represent two novel tubulin subfamilies in the eukaryotic clade of tubulins. In global phylogenetic analyses of the FtsZ/Tubulin superfamily, BtubA and BtubB stably clustered within the clade of eukaryotic tubulin subfamilies (i.e., the Tubulin family). A second stable group of sequences comprised bacterial and archaeal tubulin homologues (FtsZ, FtsZ-like, TubZ, RepX). The relationships between tubulin subfamilies were instable (except β-θ and α-κ). Here and in further phylogenetic analyses (Figure S11, Tables S1 and S2, and Materials and Methods) no stable associations between BtubA or BtubB and any tubulin subfamily were detected, in agreement with a previous less comprehensive study [11]. Shown is one representative maximum likelihood tree calculated using a 10% minimum similarity filter. A black circle indicates that the respective node/group was stable in different trees. Bar represents 1% estimated evolutionary distance. Numbers indicate how many sequences were included in a closed group.

**Figure 7:**
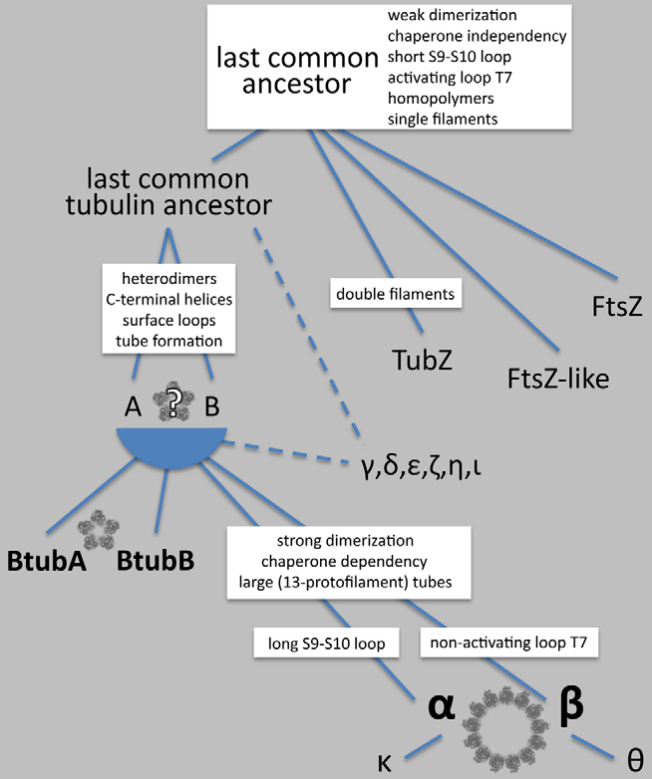
Model for the evolution of BtubA/B. Tubulins, FtsZ, FtsZ-like, and TubZ all evolved from a common ancestor with the likely properties listed [5],[9],[58]–[61]. In contrast to the bacterial FtsZ, FtsZ-like, and TubZ proteins, the last common tubulin ancestor appears to have evolved to form heterodimers (consisting of “A”- and “B”-tubulins) with properties that enabled tube formation. Modern α- and β-tubulin further localized the activating T7 and short S9, S10 loop into different subunits, developed a need for chaperones, and began to form larger, ∼13-protofilament microtubules. In contrast, BtubA and BtubB retained ancient features shared by FtsZ such as chaperone independence, weak dimerization, and both an activating T7 loop and short S9, S10 loop in both subunits [17],[19],[21]. The smaller, five-protofilament, one-start-helical architecture of the bacterial microtubule is therefore likely a primordial form. The ancestry of the other supplemental tubulins γ through κ is unclear, except that θ- and κ-tubulins derived from β and α, respectively.

It has been suggested that BtubA and BtubB evolved from modern eukaryotic α- and/or β-tubulins [11],[17],[19],[20]. If this were true, a phylogenetic association linking BtubA and BtubB to α- and/or β-tubulin would be expected. As shown previously [11],[12], BtubA and BtubB are clearly members of the eukaryotic clade of tubulins (Figure 6). A protein motif search (Table S1), an identity matrix (Table S2), and various treeing methods (Figure 6, Figure S11), however, all failed to detect any stable associations between BtubA or BtubB with any eukaryotic tubulin subfamily. BtubA and BtubB should therefore be considered as two novel tubulin subfamilies, derived not from any particular modern subfamily but instead directly from ancient tubulins. This hypothesis (Figure 7) also seems more probable because, like FtsZ but unlike eukaryotic tubulins, BtubA and BtubB exhibit the presumably ancient properties of folding without chaperones and forming weak dimers [17],[19],[20]. Furthermore, BtubA/B polymerizes in broader conditions and both proteins have mixtures of the structural characteristics found in α- and β-tubulin (activating T7 and short S9, S10 loops) [17],[21]. It therefore appears that in tubulin evolution, heterodimer formation correlated with tube formation and the five-protofilament, one-start helix was the simplest and earliest microtubule architecture realized, which later evolved into the larger eukaryotic microtubule.

While BtubA/B likely represent an ancient form of tubulin, the origin of the genes found today in *Prosthecobacter* remains unclear. The appearance of the *btubA*, *btubB*, and *bklc* genes as a distinct bacterial operon inserted in the midst of functionally related genes, but in different places in the chromosomes in the three species concerned, still points to horizontal gene transfer [18]. The lack of relatedness of BtubA/B to other tubulin families, however, makes clear that it was not a transfer from a modern eukaryote. Instead, it may have been from a yet-unidentified bacterial lineage that also carries the *btubAB* genes. The alternative, “vertical evolution” hypothesis is that *btubAB* was present in the last common ancestor of *Verrucomicrobia*, but the genes were simply lost by the other members of the phylum. It is presently debated whether an ancient *Planctomycetes*-*Verrucomicrobia*-*Chlamydiae* bacterium was involved in the evolution of eukaryotes [15],[31],[32], but if so, such a relationship would be consistent with bMTs preceding modern eukaryotic MTs.

Because eukaryotic tubulins require chaperones and accessory proteins to fold and function properly, cell biological studies and anti-microtubule drug screenings typically require that tubulin be purified from tissue. BtubA/B, however, is more stable, can be easily mutated [20],[21], recombinantly expressed in *E. coli* [17],[19]–[21], and as shown here, polymerized into microtubules in vitro. bMTs or eukaryotized derivatives could therefore complement eukaryotic microtubules as models and tools for tubulin research.

## Materials and Methods

### *Prosthecobacter* Strains and Culture

Cultures of *Prosthecobacter debontii* DSM14044, *Prosthecobacter vanneervenii* DSM12252, *Prosthecobacter dejongeii* DSM12251, and *Prosthecobacter fluviatilis* KCTC22182 were grown aerobically in DSM medium 628. The cultures were incubated static at 20°C or shaking (200 rpm) at 30°C.

### Reverse Transcriptase-Polymerase Chain Reaction (RT-PCR)

Total RNA was purified from cultures using the TRIzol Reagent (Invitrogen) and RNA was subsequently treated with RQ1 RNase-free DNase (Promega). First strand cDNA was synthesized using RevertAid M-MuLV reverse transcriptase and random hexamer primers (RevertAid First Strand cDNA Synthesis Kit, Fermentas). A negative control was run without reverse transcriptase enzyme. Fragments of *btubA* or *btubB* were PCR-amplified from the cDNA using specific primers. PCR-reactions were analyzed by agarose gel electrophoresis.

### PCR Screening of *P. fluviatilis* for Tubulin Genes

Primers [12] targeting conserved tubulin sequences were used to PCR-amplify potential tubulin genes from genomic *P. fluviatilis* DNA.

### Heterologous Gene Expression

Genomic *Prosthecobacter* DNA was isolated according to Wisotzkey et al. [33]. *btub*-operon genes from *P. vanneervenii* or *P. dejongeii* were amplified by PCR using PfuUltra polymerase (Stratagene), generating fragments with unique restriction sites at the 5′-end (NdeI) and the 3′-end (BamHI or EcoRI). After digestion, the PCR fragments were cloned into a digested vector derived from pHis17. pHis17 was provided by D. Schlieper/J. Löwe [17]. Plasmid inserts were verified by sequencing. For protein expression, plasmids were transformed into *Escherichia coli* C41(DE3) cells [34]. The proteins were expressed under control of the T7 promoter. Depending on the restriction enzymes used (NdeI-BamHI or NdeI-EcoRI) and PCR primer design, eight residues were added to the C-terminus of the expressed protein (GSHHHHHH or EFHHHHHH, respectively). Typically, cells were cultured overnight at 37°C in LB-amp medium (10 g tryptone, 5 g yeast extract, and 10 g NaCl per liter of water; 50 µg/ml ampicillin), cultures were diluted 10-fold in LB-amp, cells were incubated for 1 h, and expression was induced by addition of 1 mM isopropyl–D-thiogalactopyranoside (IPTG).

### Protein Purification

C-terminally His-tagged *P. dejongeii* BtubA and BtubB were expressed in *E. coli* C41(DE3). After 3-h induction (30°C, 1 mM IPTG), cells were lysed in buffer A (20 mM Tris-HCL, 300 mM NaCl, 40 mM imidazole, pH 7.5) using a microfluidizer. Cell walls and insoluble debris was removed by centrifugation (4°C, 30 min, 50 k×g). The supernatant was loaded on a Ni-NTA affinity chromatography column (HisTrap FF, GE Healthcare). The column was washed with buffer A and the proteins were eluted using buffer A containing 250 mM imidazole. Proteins were dialyzed into PBS (0.14 M NaCl, 2.7 mM KCl, 10.1 mM Na_2_HPO_4_, 1.8 mM KH_2_PO_4_, pH 7.4). Before freezing and storage GTP was added at equal concentrations to BtubA and BtubB, respectively [19].

### BtubA/B in vitro Polymerization

BtubA/B was polymerized by a similar method to that described by Sontag et al. [19]. Purified BtubA and BtubB were equilibrated into HMK buffer (50 mM Hepes, 5 mM MgAc, 350 mM KAc, 1 mM EGTA, pH 7.7) using buffer exchange spin columns (Pierce). For plunge-freezing, BtubA (25 µM) and BtubB (25 µM) were polymerized with 1 mM GTP in a 50 µl volume at 20°C for 15 min. For high-pressure freezing, BtubA (65 µM) and BtubB (65 µM) were polymerized with 1 mM GTP in a 50 µl volume at 20°C for 30 min.

### Western Hybridization

Cells were mixed with SDS-PAGE loading buffer, separated on an SDS-PAGE gel, and proteins were transferred onto a PVDF membrane (Pall). The membrane was blocked (4°C, 12 h) in PBS-T (0.14 M NaCl, 2.7 mM KCl, 10.1 mM Na_2_HPO_4_, 1.8 mM KH_2_PO_4_, 0.1% (v/v) Tween 20, pH 7.4) supplemented with 5% fat-free dry milk, incubated (1 h, 20°C) with the primary antibody (diluted in PBS-T; anti-BtubA 1/70,000, anti-BtubB 1/60,000), washed in PBS-T, incubated (1 h, 20°C) with peroxidase-conjugated secondary anti-IgG antibody (diluted 1/70,000 in PBS-T; Pierce), and washed in PBS-T. The signal was detected using ECL Western Blotting Substrate (Pierce) and X-ray films. Polyclonal rabbit anti-BtubA and anti-BtubB antibodies were kindly provided by H. Erickson [19].

### Conventional Electron Microscopy

For negative staining, samples were applied to a Formvar-coated, carbon-coated, glow-discharged copper EM grid (Electron Microscopy Sciences). Samples were aspirated and stained with 0.5%–2% uranylacetate.

For thin-section EM, cells were pelletted and resuspended in growth medium containing 10% Ficoll (70 kD). Cells were pelletted again, transferred to aluminum planchettes, and high-pressure frozen in a Bal-Tec HPM-010 (Leica Microsystems). The frozen cells were transferred to a AFS Freeze-Substitution machine (Leica) and freeze-substituted into 2% or 0.04% glutaraldehyde in acetone at −90°C for 60 h, then warmed to −20°C over 10 h. Cells were rinsed 3× with cold acetone, then post-fixed with 2.5% osmium tetroxide in acetone at −20°C for 24 h. The samples were then warmed to 4°C over 2 h, rinsed 3× with cold acetone, and embedded in Epon-Araldite resin (Electron Microscopy Sciences). Following polymerization, semi-thin (200 nm) sections were cut with a UC6 ultramicrotome (Leica), stained with uranyl acetate and lead citrate, and imaged in a Tecnai T12 TEM (FEI). Tomographic tilt-series were acquired using the SerialEM [35] software package, then subsequently calculated and analyzed using IMOD [36].

### Immuno-Electron Microscopy

Exponentially growing cells were prepared for immuno-EM by a modification of the method of Tokuyasu [37],[38]. Briefly, cells were fixed with PBS containing 4% paraformaldehyde (Electron Microscopy Sciences) and 5% sucrose for 8 h at 4°C. The cells were then pelleted and infiltrated with 2.1 M sucrose in PBS over 12 h. Pellets were transferred to aluminum sectioning stubs (Ted Pella, Inc.) and quickly frozen in liquid nitrogen. Thin cryo-sections (90 nm) were cut at −110°C with an EM-UC6/FC6 cryo-ultramicrotome (Leica Microsystems) using a cryo-diamond knife (Diatome). Sections were transferred to Formvar-coated, carbon-coated, glow-discharged 200-mesh copper/rhodium EM grids (Electron Microscopy Sciences) and labeled with anti-BtubB antibodies (kindly provided by H. Erickson [19]; diluted 1/5,000), then gold (10 nm) conjugated anti-rabbit secondary antibodies (Ted Pella, Inc). Sections were negatively stained with 1% uranyl acetate and stabilized with 1% methylcellulose. Samples were imaged with a Tecnai T12 electron microscope (FEI Company).

### Cryosectioning

In vitro BtubA/B-polymerization reactions were supplemented with an equal volume of a suspension of colloidal gold (10 nm) and dextran (40% w/v) in HMK. Pelleted cells were spun through a cryo-protectant solution (20% dextran w/v in culture medium). The samples were transferred to brass planchettes and rapidly frozen in a high-pressure freezing machine (Bal-Tec HPM-010, Leica Microsystems). Cryosectioning of the vitrified samples was done as previously described [39],[40]. Semi-thin (90–130 nm) cryosections were cut at −145°C or −170°C with a 25° or 35° Cryo diamond knife (Diatome), transferred to grids (continuous-carbon coated 200-mesh copper grids or 700-mesh uncoated copper grids), and stored in liquid nitrogen.

### Plunge-Freezing

For plunge-freezing, copper/rhodium EM grids (R2/2 or R2/1, Quantifoil) were glow-discharged for 1 min. A 20×-concentrated bovine serum albumin-treated solution of 10 nm colloidal gold (Sigma) was added to the bacterial culture or polymerization reaction (1∶4 v/v) immediately before plunge freezing. A 4-µl droplet of the mixture was applied to the EM grid, then automatically blotted and plunge-frozen into a liquid ethane-propane mixture [41] using a Vitrobot (FEI Company) [42]. Alternatively, EM grids were incubated in a static liquid culture, removed, blotted, and plunge-frozen. The grids were stored in liquid nitrogen.

### Cryo-Electron Microscopy

Cryo-EM images were collected using a Polara 300 kV FEG transmission electron microscope (FEI Company) equipped with an energy filter (slit width 20 eV; Gatan) on a lens-coupled 4 k×4 k UltraCam (Gatan). Pixels on the CCD represented 0.95 nm (22,500×), 0.63 nm (34,000×), or 0.51 nm (41,000×) at the specimen level. Typically, tilt series of whole cells were recorded from −60° to +60° with an increment of 1° at 10 µm under-focus. Tilt series of in vitro polymerized proteins were recorded from −69° to +69° with an increment of 1–2° at 6–12 µm under-focus. The cumulative dose of a tilt-series was 180–220 e^−^/Å^2^ (for whole cells) or 80–100 e^−^/Å^2^ (for in vitro polymerized proteins). Leginon [43] or UCSF Tomo [44] was used for automatic tilt-series acquisition. Three-dimensional reconstructions were calculated using the IMOD software package [36] or Raptor [45].

### Sub-Tomogram Averaging

The averaging procedure described by Cope et al. [46] was adapted to bacterial tubulin structures. IMOD [36] was used to correct selected tomograms for the CTF and to model the center of the tube. The program addModPts was run to fill in model points every 4.5 nm or 9 nm along the tube axis (for averaging overlapping sub-volumes). Alternatively, model points were set manually at a distance of 42 nm or 21 nm (for averaging unique sub-volumes). The PEET software package [47] was used to align and average repeating sub-volumes. Isosurface rendering of the sub-volume averages was carried out using IMOD [36].

### Construction of Pseudo-Atomic Model

BtubA/B coordinates (2 btq) were arranged using UCSF Chimera [48]. The 15°-intradimer bend seen in crystals [17] was straightened and heterodimers were replicated and shifted 8.8 nm to generate a protofilament consisting of six heterodimers. The protofilament was replicated and tubes were built using four, five, or six protofilaments, each shifted 0.88 nm with respect to the previous protofilament along the tube axis. The 5-nm protofilament spacing seen in eukaryotic microtubules seemed unreasonable in these much smaller-diameter tubes, since inter-protofilament interactions appeared impossible. Protofilaments were therefore brought closer together (4.6 nm) to best allow lateral interactions.

### Tomogram Simulation

The tomogram simulation procedure described by Gan et al. [49] was adapted to bacterial tubulins. All simulations were done with Bsoft [50] using imaging parameters close to the nominal experimental conditions. Briefly, a 3-D map was generated with a 0.096-nm voxel and then projected to create a tilt series with ±60° total tilt in 1° increments. Images were then multiplied in reciprocal space with a 10-µm underfocused CTF and then re-sampled using a pixel-size of 0.96 nm. Tomograms were reconstructed with IMOD [36] using the same settings as for the experimental data.

### Segmentation of Tomogram Components

The Slicer-tool in 3dMOD was used to orient tomograms, present 2-D slices, and produce series of TIF images. TIF images were combined to form a new volume using IMOD [36]. Tubes and other cell components in the tomogram were then segmented manually using Amira (Visage Imaging GmbH).

### Diffraction Patterns

ImageJ was used to calculate Fourier transforms of BtubA/B tubes in 2-D projection images, 2-D slices through tomograms, or simulated 2-D images. Subunit repeat distances in *Prosthecobacter*, *E. coli*, and in vitro bMTs were estimated from layer line positions.

### Phylogenetic Sequence Analyses

Protein sequences were analyzed using the program PRINTS [51] in order to detect shared motifs and calculate a probability value for the likelihood that different BtubA or BtubB proteins belonged to a particular tubulin family. To perform phylogenetic sequence analyses, two different databases were established using the ARB program package [52]. The two databases, Tubulin_ClustalW and Tubulin_Conserved_Domain, contained 240 entries which represented bacterial and archaeal FtsZs, different eukaryotic tubulin subfamilies, BtubA and BtubB, *Bacillus* Tubulin-likes, and archaeal FtsZ-likes. For the Tubulin_ClustalW database, amino acid sequences were first aligned using ClustalW [53]. For the Tubulin_Conserved_Domain database, sequences were aligned according to the tubulin alignment available at the Conserved Domain Database [54]. In both databases, the amino acid alignments were refined manually accounting for conserved tubulin domains. The identity matrix for a selection of representatives was generated using the ARB program package [52].

For tree calculations, two filters were produced (prot_10, prot_30), each retaining only positions conserved in at least 10% or 30% of the selected sequences, respectively. Phylogenetic analyses were performed using distance matrix methods (programs ARB neighbor joining and Phylip UPGMA, FITCH), maximum parsimony (program Phylip PROTPARS), and maximum likelihood (programs Phylip PROML, PHYML, and TREE-PUZZLE). All programs are implemented in the ARB program package [52]. Each analysis was repeated using the different treeing methods in combination with both filters. For the TREE-PUZZLE method a smaller selection of sequences was used due to calculation time limits. Neighbor joining and maximum parsimony trees were bootstrap re-sampled (1,000 and 100 bootstraps, respectively) and the number of puzzling steps for TREE-PUZZLE trees was 1,000. For distance matrix methods and the maximum likelihood method the Dayhoff PAM matrix substitution model was used. For the TREE-PUZZLE method the Muller-Vingron substitution model [55] was used. The topologies of all trees were compared to recover the most stable associations.

## Supporting Information

Figure S1 *btubA/btubB* genes are transcribed in *P. vanneervenii* cultures. *btubA*- and *btubB*-mRNAs were detected by reverse transcription of total mRNA isolated from cultures and specific PCR-amplification of *btubA*- or *btubB*-fragments from cDNA. Reactions were run in duplicates. (+RT) PCR-amplification from total mRNA reversely transcribed into cDNA; (-RT) PCR-amplification from a control sample processed without reverse transcriptase; (C) control PCR sample processed without template. Gene names indicate which gene was specifically amplified during PCR; numbers indicate base-pair lengths of DNA standard.
(PDF)

Figure S2 BtubA and BtubB proteins are present in *Prosthecobacter*. Western blots for BtubB (left) and BtubA (right) proteins are shown for *btubAB*-harboring *Prosthecobacter* strains [11],[12] *P. debontii* (Pdb), *P. dejongeii* (Pdj), and *P. vanneervenii* (Pva), and the *btubAB*-lacking strain [24] *P. fluviatilis* (Pfl). Anti-BtubA antibodies appear to bind BtubA in Pdb, BtubB in Pdj, and both BtubA and BtubB proteins in Pva. Numbers indicate standard protein size in kDa.
(PDF)

Figure S3 Additional examples of bacterial microtubules in prosthecobacters. 11.4-nm tomographic slices (top) with cell overviews (top inset; rectangle indicates imaged region), and enlarged views (below) of several additional bacterial microtubules (arrows) are shown, observed in different *Prosthecobacter* species (*P. dejongeii*, (A, D); *P. vanneervenii*, (B, C, E–G)). Tubes occurred (A–C) individually or in bundles of (D, E) two, (F) three, or (G) four. To visualize bundles in 3-D, BtubA/B tubes were manually segmented and colored differently. Two views of the segmentation are shown in panels E–G (bottom). See also Movie S1 for additional views of a bundle of four tubes. Scale bars are 100 nm.
(PDF)

Figure S4 BtubA/B-structures are not preserved well by conventional EM methods. Samples were prepared by the best available conventional EM methods: samples were high-pressure frozen, freeze-substituted (dehydrated, fixed, and stained at low temperature), plastic-embedded, thin sectioned, and imaged by tomography. No cytoskeletal structures were seen in *P. vanneervenii* (A). *E. coli* cells expressing BtubA/B frequently showed “ghost-like” structures (arrows) that are presumably remnants of bacterial microtubules (B). The sample with in vitro polymerized BtubA/B only showed poor resolution in both views, longitudinal (upper) and perpendicular (lower). Similar low-resolution images have been published previously (Figure 2 in [19]). Only a special protocol (0.04% instead of 2% glutaraldehyde) yielded in visible filamentous structures in *E. coli* cells expressing BtubA/B (D). Scale bars are 100 nm.
(PDF)

Figure S5 Localization of BtubB in *P. vanneervenii*. BtubB proteins were localized by immuno-EM staining with primary anti-BtubB antibodies and 10-nm gold-labeled secondary antibodies. Specific signals (arrows) were found mainly in the stalk or in the transition zone between cell body and stalk, matching the positions of the tubes in the cryo-tomograms. Scale bar is 600 nm.
(PDF)

Figure S6 Localization of BtubB in *P. fluviatilis* – negative control. As a negative control, *P. fluviatilis* cells (which lack *btubA/B* genes) were searched for BtubB by immuno-EM staining with primary anti-BtubB antibodies and 10-nm gold-labeled secondary antibodies. No specific signals were detected, verifying the specificity of the approach used for *P. vanneervenii* (Figure S5). Scale bar is 600 nm.
(PDF)

Figure S7 Simulated tomograms of modeled bacterial microtubules. In the experimental tomograms of bacterial microtubules, a left-right asymmetry was frequently observed (Figure 3A–C). To investigate if such an asymmetry might have arisen because of an odd number of protofilaments, tomograms were simulated of four-, five-, and six-protofilament tubes lying perpendicular to the electron beam and parallel to the tilt axis but with different rotations around the tube axis (as indicated by the schematics). 11.4-nm thick slices through the simulated tomograms are shown. Only the five-protofilament tubule results in left-right asymmetry (indicated by arrows), supporting the notion that bacterial microtubules contain five protofilaments. Scale bar is 10 nm.
(PDF)

Figure S8 Sub-tomogram averaging of bacterial microtubules. The isosurface of a sub-tomographic average of a bacterial microtubule within a *Prosthecobacter vanneervenii* cell is shown from different angles as indicated in the left panel. Left/right asymmetry is clearly visible from the side views (3 and 4), as outlined by the distance between the parallel red lines. The consistent asymmetry seen here and further along the tube (not shown) suggests that the five protofilaments in the bacterial microtubule are straight, since the maximum rotation angle permitted during the alignment of sub-tomograms was restricted to ±15°. If the protofilaments had been twisting around the tube, the asymmetry would have been averaged out. Scale bar is 10 nm.
(PDF)

Figure S9 Projection images of negatively stained BtubA/B tubes polymerized in vitro. Samples with low protein concentrations (1.2 µM each) or samples analyzed at early time points (30 s–1 min), respectively, frequently showed pairs of parallel densities ∼7.6 nm apart (A). Later (5 min–1 h) or at higher protein concentrations (5 µM each), respectively, longer pairs were seen aligned in bundles (B). Similar images have been published previously [17],[19], but the structures were interpreted as protofilament bundles. Given our knowledge that the proteins form tubes in vivo with similar dimensions, we believe the parallel lines represent the walls of bacterial microtubules rather than protofilament pairs. In some images, the structures stained positively, further revealing their tubular nature (C). Bars, 50 nm.
(PDF)

Figure S10 Fourier transforms of simulated projections of BtubA/B tube models with different rotations. Projection images of one-start helical models of BtubA/B tubes were simulated and Fourier transformed. The spots on the subunit repeat layer line (arrowheads) were asymmetric in all cases, but the asymmetry changed depending on the rotation on the tube around its length axis (angles indicated). Since asymmetry was detected in both “B-lattice and seam” and “A-lattice without seam” tubes, the asymmetry seems to arise from the small number of protofilaments (and resulting lack of an extended “front” and ”back” side) and not from the presence of a seam.
(PDF)

Figure S11 Phylogenetic relationships within the Tubulin family. Consensus tree showing only stable associations recovered in the majority of individual trees. The tubulin subfamilies α, β, γ, ε, and the groups BtubA and BtubB were recovered as monophyletic groups with relatively high support. δ-tubulins were sometimes split in two groups. With two exceptions (α-κ and β-θ), no specific associations between any of the tubulin subfamilies could be detected. Previous, less comprehensive studies made similar observations [11],[56],[57]. Likewise, BtubA and BtubB showed neither a special relationship between themselves nor an association to any tubulin subfamily and should therefore be considered as individual, novel tubulin subfamilies. Because duplication and evolution (θ and κ) of modern tubulins (β and α) are clear, the analyses do not support the hypothesis that BtubA and BtubB derived from modern α- and/or β-tubulins. The consensus is of 28 trees produced using two different alignments, seven treeing algorithms, and two different filters (Materials and Methods). Support values for six trees of the Tubulin_ClustalW database are reported at the branches, from left to right: maximum parsimony (100 bootstraps)/neighbor joining (1,000 bootstraps)/TREE-PUZZLE (1,000 puzzling steps) with a 30% (upper numbers) or 10% minimum similarity filter (lower numbers). The asterisk denotes a node, which was not recovered in the respective tree. Numbers within closed groups refer to the number of included sequences; due to calculation limits TREE-PUZZLE trees were calculated using a reduced number of sequences (number in parentheses).
(PDF)

Table S1 BtubA and BtubB protein motif search. PRINTS [51] was used to identify defining motifs of tubulin-related proteins (all tubulins, alpha, beta, gamma, delta, epsilon, and FtsZ) in four different BtubA and BtubB proteins (from *P. dejongeii*, *P. vanneervenii*, *P. debontii* operon 1, and *P. debontii* operon 2). The chart lists the number of motifs a protein shares with each group, as well as the “P-value” (the probability that a random sequence would achieve a higher score). BtubA and BtubB are clearly more similar to eukaryotic tubulin than to bacterial FtsZ, but they do not belong to any particular eukaryotic tubulin subfamily (as a control, the P-value of human α-tubulin with the α-tubulin subfamily is 10^−121^).
(PDF)

Table S2 Sequence identities within the Tubulin/FtsZ superfamily. BtubA and BtubB share the highest sequence identity with eukaryotic tubulin subfamilies, but no clear relationship of BtubA or BtubB to any specific tubulin subfamily (shaded in grey) or between BtubA and BtubB could be detected. Identity values are in percentages. Pva, *P. vanneervenii*; Pdb, *P. debontii*; Pdj, *P. dejongeii*; Pte, *Paramecium tetraurelia*; Ddi, *Dictyostelium discoideum*; Hsa, *Homo sapiens*; Bth, *Bacillus thuringiensis*; Bce, *Bacillus cereus*; Hal, *Halobacterium* species.
(PDF)

Movie S1 Bundling pattern of four bacterial microtubules. Bacterial microtubules were observed alone or in bundles of up to four. The movie shows a bundle of four parallel bacterial microtubules (same bundle as shown in Figure 1B). Cross-sectional views (2-D tomographic slices) are followed by a 3-D segmentation (the four different bacterial microtubules are shown in four different colors).
(MOV)

## Abbreviations

bMT: bacterial microtubule
BtubA: bacterial tubulin A
BtubB: bacterial tubulin B
ECT: electron cryotomography
EM: electron microscopy
MT: microtubule
