# Supplementary figures and images for: Microtubules in Bacteria: Ancient Tubulins Build a Five-Protofilament Homolog of the Eukaryotic Cytoskeleton
Source: PLoS Biol. 2011 Dec 6;9(12):e1001213. doi: 10.1371/journal.pbio.1001213 (PMC3232192; doi:10.1371/journal.pbio.1001213)

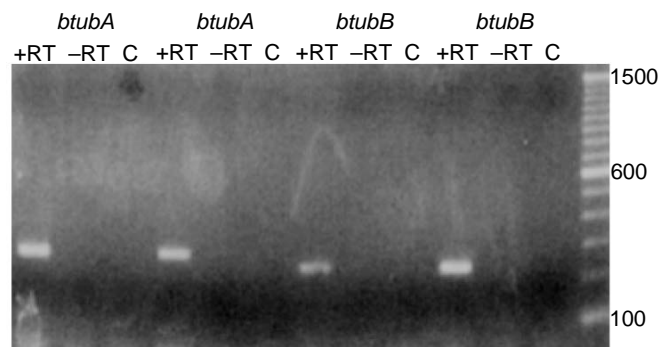

**Figure S1. *btubA/btubB* genes are transcribed in *P. vanneervanii* cultures.**

Supplement: Figure S1 — btubA/btubB genes are transcribed in P. vanneervenii cultures. btubA- and btubB-mRNAs were detected by reverse transcription of total mRNA isolated from cultures and specific PCR-amplification of btubA- or btubB-fragments from cDNA. Reactions were run in duplicates. (+RT) PCR-amplification from total mRNA reversely transcribed into cDNA; (-RT) PCR-amplification from a control sample processed without reverse transcriptase; (C) control PCR sample processed without template. Gene names indicate which gene was specifically amplified during PCR; numbers indicate base-pair lengths of DNA standard. (PDF) [file pbio.1001213.s001.pdf]

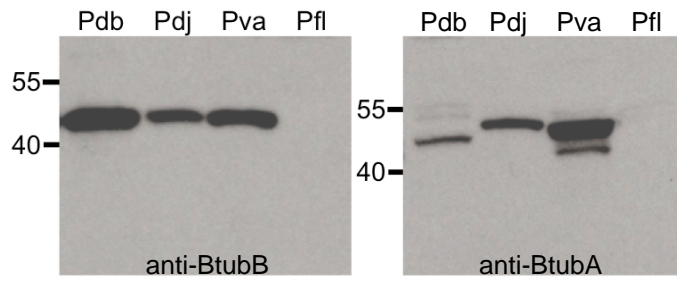

**Figure S2. BtubA and BtubB proteins are present in *Prosthecobacter*.**

Supplement: Figure S2 — BtubA and BtubB proteins are present in Prosthecobacter. Western blots for BtubB (left) and BtubA (right) proteins are shown for btubAB-harboring Prosthecobacter strains [11],[12] P. debontii (Pdb), P. dejongeii (Pdj), and P. vanneervenii (Pva), and the btubAB-lacking strain [24] P. fluviatilis (Pfl). Anti-BtubA antibodies appear to bind BtubA in Pdb, BtubB in Pdj, and both BtubA and BtubB proteins in Pva. Numbers indicate standard protein size in kDa. (PDF) [file pbio.1001213.s002.pdf]

**A**

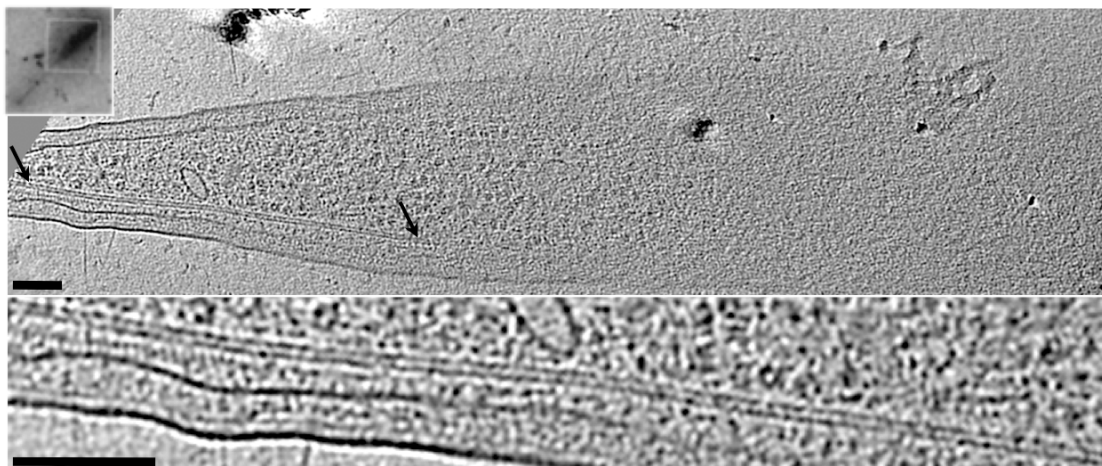

**B**

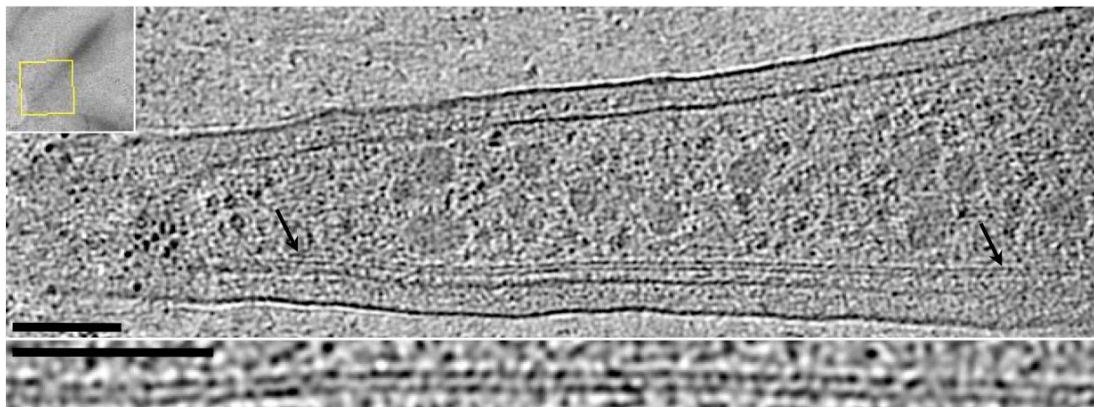

**C**

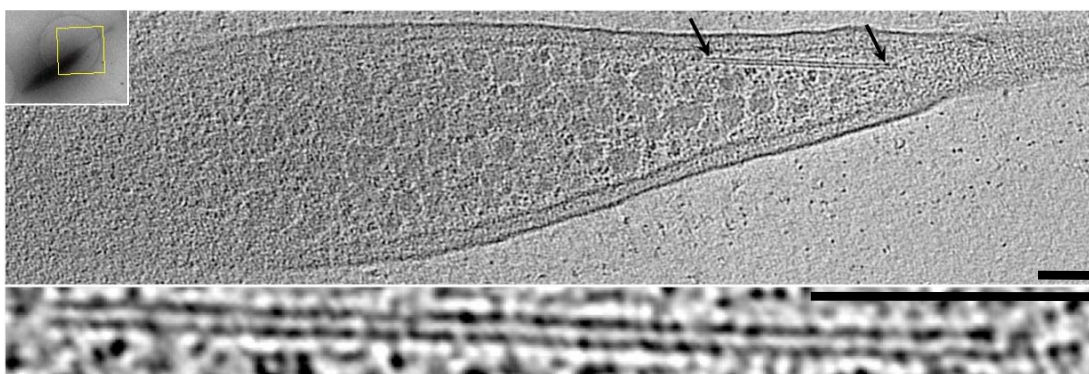

D

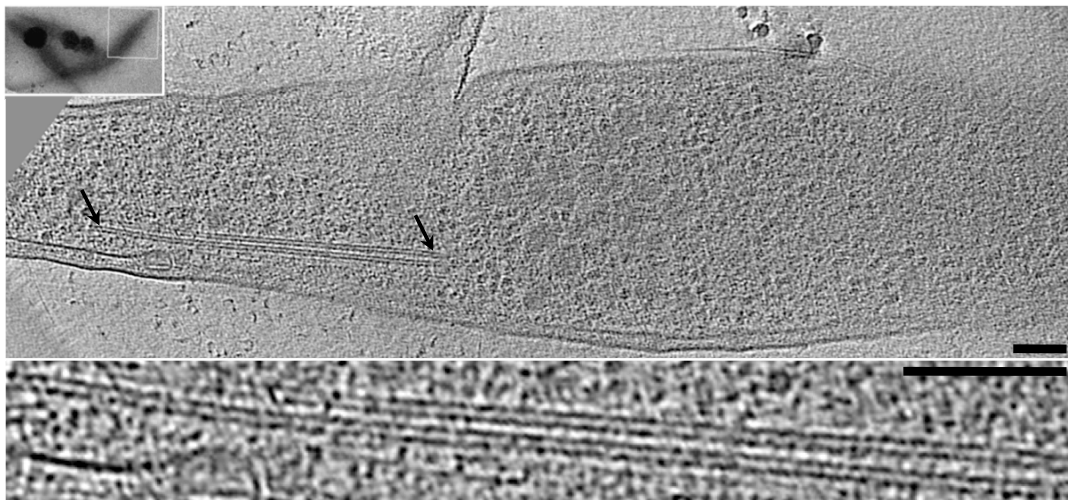

E

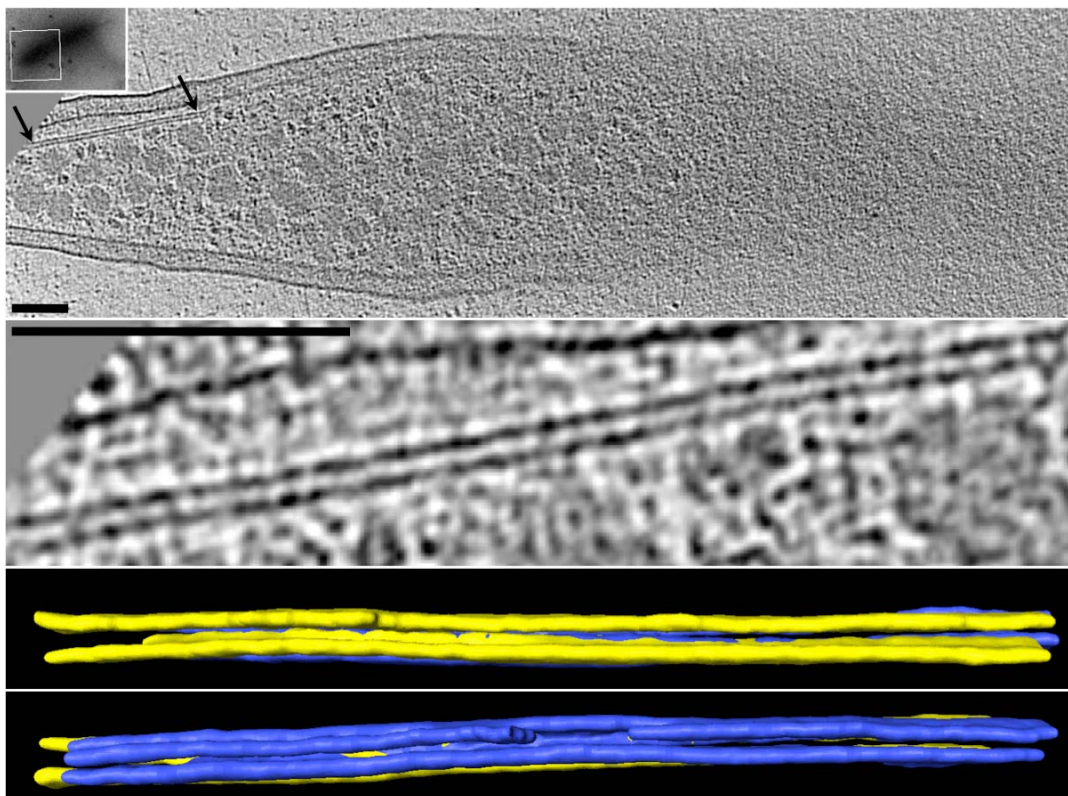

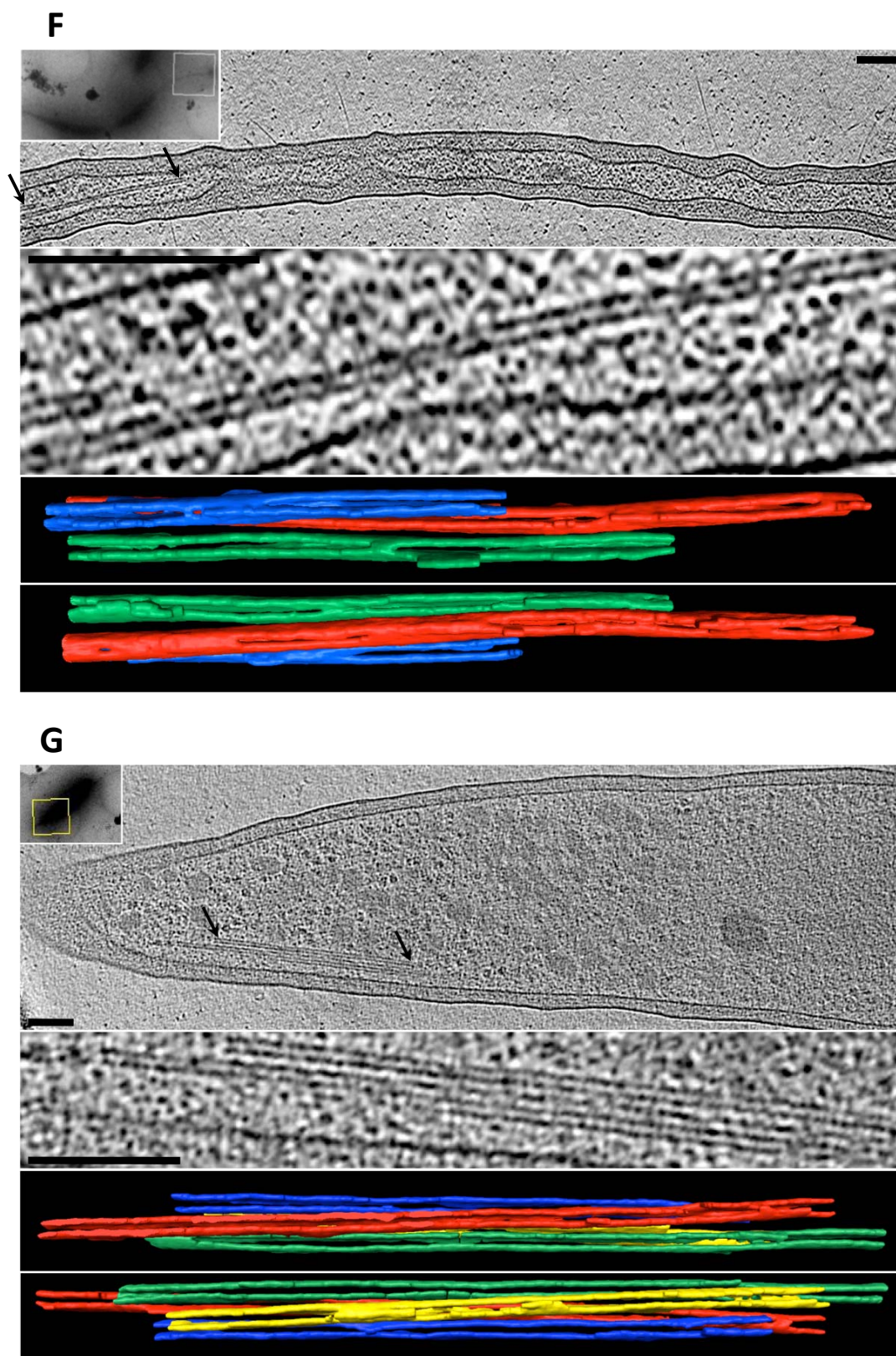

**Figure S3. Additional examples of bacterial microtubules in *Prosthecobacter*.**

Supplement: Figure S3 — Additional examples of bacterial microtubules in prosthecobacters. 11.4-nm tomographic slices (top) with cell overviews (top inset; rectangle indicates imaged region), and enlarged views (below) of several additional bacterial microtubules (arrows) are shown, observed in different Prosthecobacter species (P. dejongeii, (A, D); P. vanneervenii, (B, C, E–G)). Tubes occurred (A–C) individually or in bundles of (D, E) two, (F) three, or (G) four. To visualize bundles in 3-D, BtubA/B tubes were manually segmented and colored differently. Two views of the segmentation are shown in panels E–G (bottom). See also Movie S1 for additional views of a bundle of four tubes. Scale bars are 100 nm. (PDF) [file pbio.1001213.s003.pdf]

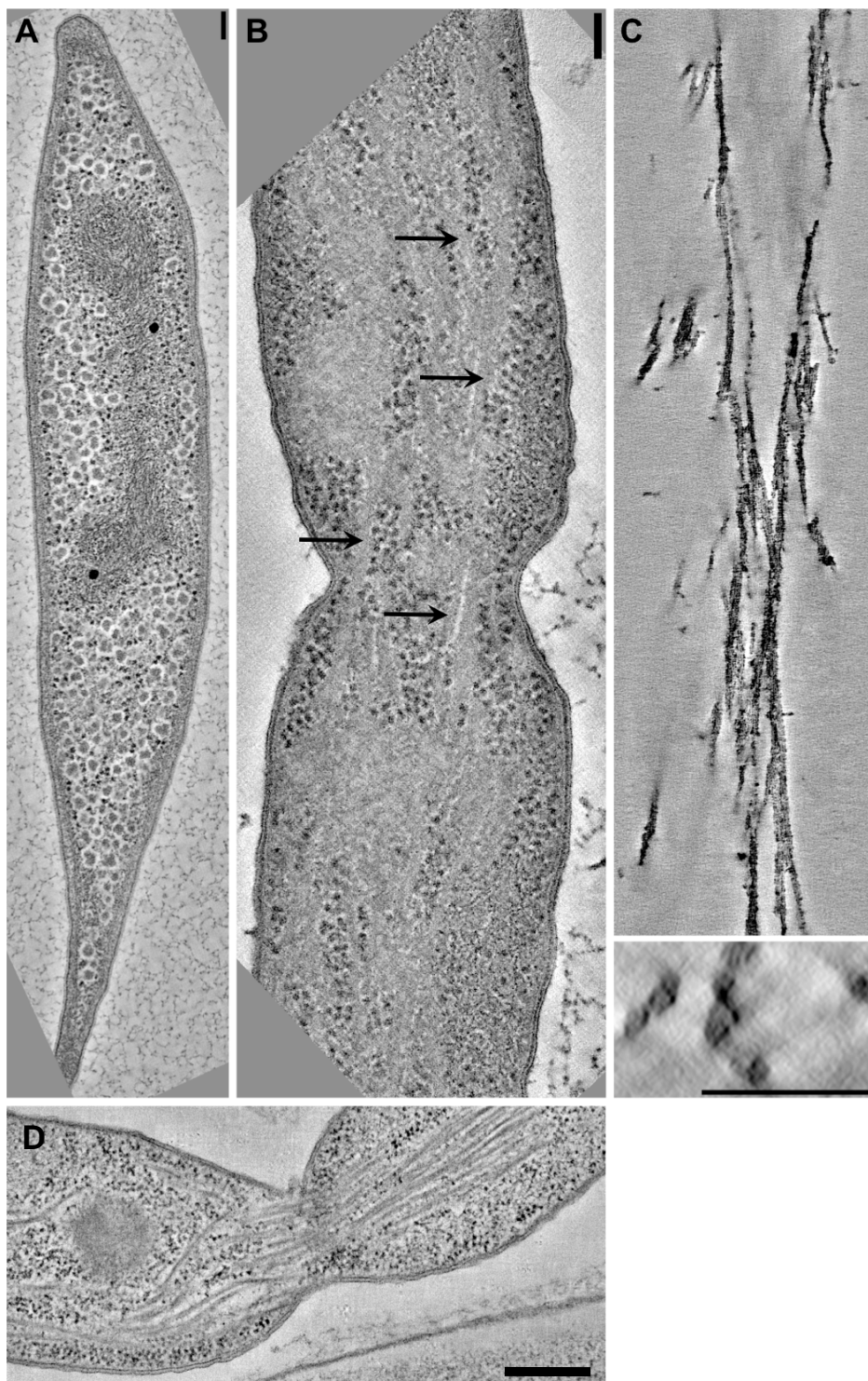

**Figure S4. BtubA/B-structures are not preserved well by conventional EM methods.**

Supplement: Figure S4 — BtubA/B-structures are not preserved well by conventional EM methods. Samples were prepared by the best available conventional EM methods: samples were high-pressure frozen, freeze-substituted (dehydrated, fixed, and stained at low temperature), plastic-embedded, thin sectioned, and imaged by tomography. No cytoskeletal structures were seen in P. vanneervenii (A). E. coli cells expressing BtubA/B frequently showed “ghost-like” structures (arrows) that are presumably remnants of bacterial microtubules (B). The sample with in vitro polymerized BtubA/B only showed poor resolution in both views, longitudinal (upper) and perpendicular (lower). Similar low-resolution images have been published previously (Figure 2 in [19]). Only a special protocol (0.04% instead of 2% glutaraldehyde) yielded in visible filamentous structures in E. coli cells expressing BtubA/B (D). Scale bars are 100 nm. (PDF) [file pbio.1001213.s004.pdf]

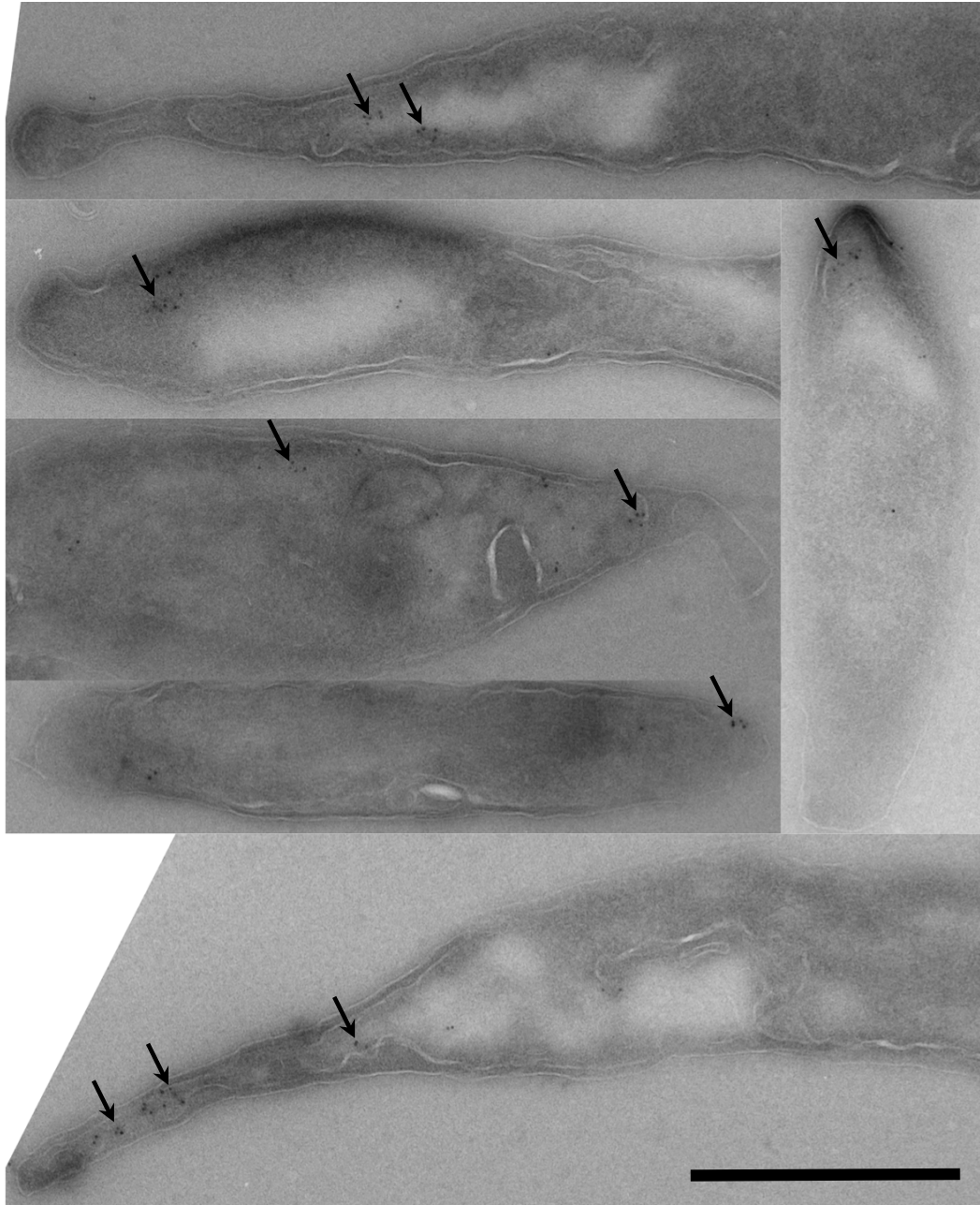

**Figure S5. Localization of BtubB in *P. vanneervanii*.**

Supplement: Figure S5 — Localization of BtubB in P. vanneervenii. BtubB proteins were localized by immuno-EM staining with primary anti-BtubB antibodies and 10-nm gold-labeled secondary antibodies. Specific signals (arrows) were found mainly in the stalk or in the transition zone between cell body and stalk, matching the positions of the tubes in the cryo-tomograms. Scale bar is 600 nm. (PDF) [file pbio.1001213.s005.pdf]

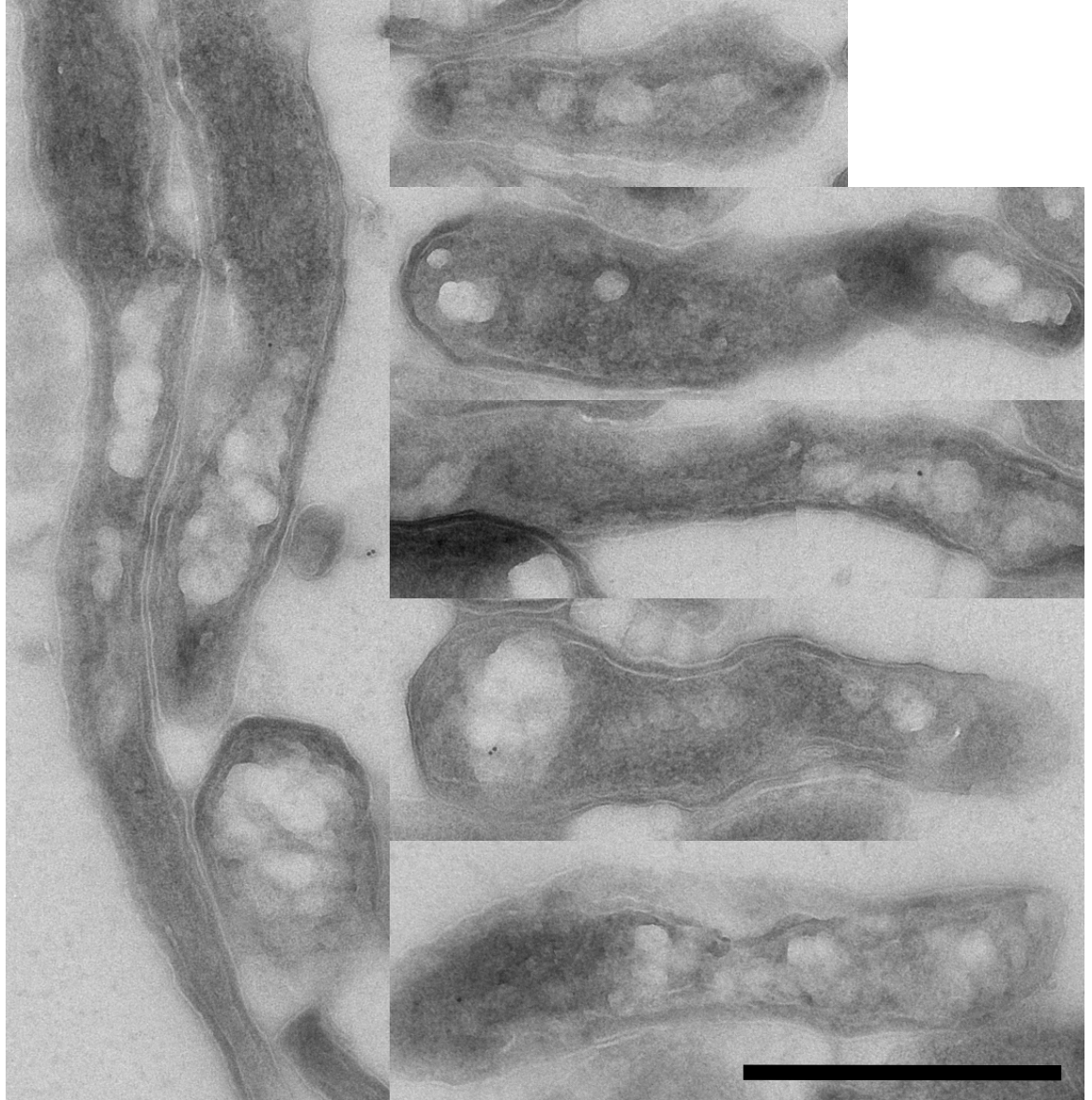

**Figure S6. Localization of BtubB in *P. fluviatilis* – negative control.**

Supplement: Figure S6 — Localization of BtubB in P. fluviatilis – negative control. As a negative control, P. fluviatilis cells (which lack btubA/B genes) were searched for BtubB by immuno-EM staining with primary anti-BtubB antibodies and 10-nm gold-labeled secondary antibodies. No specific signals were detected, verifying the specificity of the approach used for P. vanneervenii (Figure S5). Scale bar is 600 nm. (PDF) [file pbio.1001213.s006.pdf]

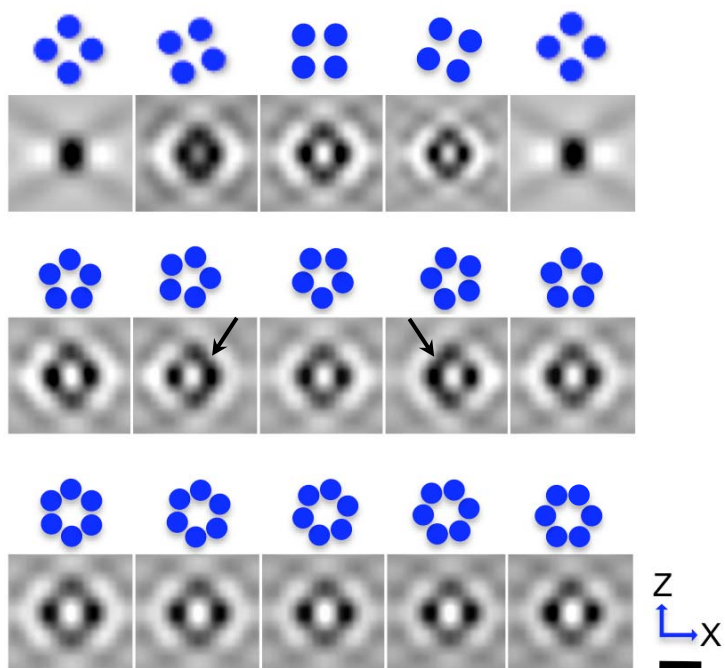

**Figure S7. Simulated tomograms of modelled bacterial microtubules.**

Supplement: Figure S7 — Simulated tomograms of modeled bacterial microtubules. In the experimental tomograms of bacterial microtubules, a left-right asymmetry was frequently observed (Figure 3A–C). To investigate if such an asymmetry might have arisen because of an odd number of protofilaments, tomograms were simulated of four-, five-, and six-protofilament tubes lying perpendicular to the electron beam and parallel to the tilt axis but with different rotations around the tube axis (as indicated by the schematics). 11.4-nm thick slices through the simulated tomograms are shown. Only the five-protofilament tubule results in left-right asymmetry (indicated by arrows), supporting the notion that bacterial microtubules contain five protofilaments. Scale bar is 10 nm. (PDF) [file pbio.1001213.s007.pdf]

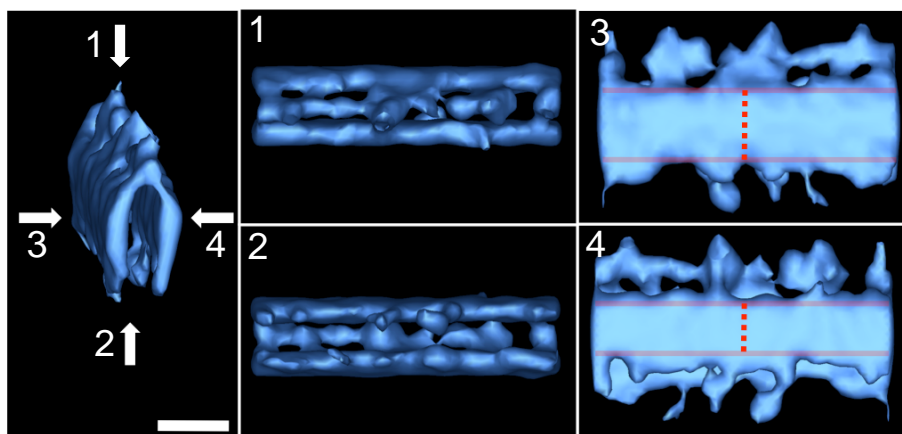

**Figure S8. Sub-tomogram averaging of bacterial microtubules.**

Supplement: Figure S8 — Sub-tomogram averaging of bacterial microtubules. The isosurface of a sub-tomographic average of a bacterial microtubule within a Prosthecobacter vanneervenii cell is shown from different angles as indicated in the left panel. Left/right asymmetry is clearly visible from the side views (3 and 4), as outlined by the distance between the parallel red lines. The consistent asymmetry seen here and further along the tube (not shown) suggests that the five protofilaments in the bacterial microtubule are straight, since the maximum rotation angle permitted during the alignment of sub-tomograms was restricted to ±15°. If the protofilaments had been twisting around the tube, the asymmetry would have been averaged out. Scale bar is 10 nm. (PDF) [file pbio.1001213.s008.pdf]

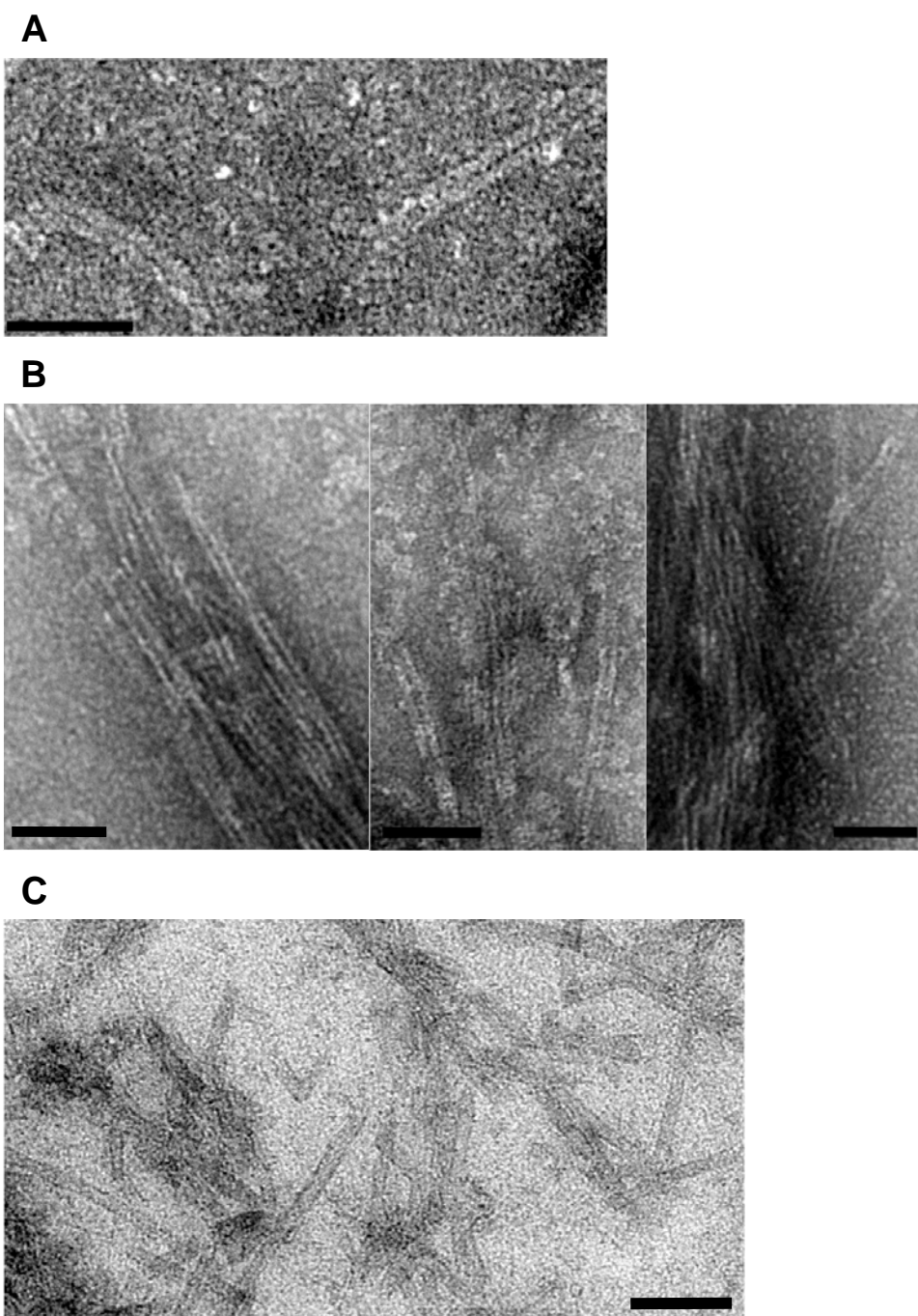

**Figure S9.** Negatively stained BtubA/B polymerized *in vitro*.

Supplement: Figure S9 — Projection images of negatively stained BtubA/B tubes polymerized in vitro. Samples with low protein concentrations (1.2 µM each) or samples analyzed at early time points (30 s–1 min), respectively, frequently showed pairs of parallel densities ∼7.6 nm apart (A). Later (5 min–1 h) or at higher protein concentrations (5 µM each), respectively, longer pairs were seen aligned in bundles (B). Similar images have been published previously [17],[19], but the structures were interpreted as protofilament bundles. Given our knowledge that the proteins form tubes in vivo with similar dimensions, we believe the parallel lines represent the walls of bacterial microtubules rather than protofilament pairs. In some images, the structures stained positively, further revealing their tubular nature (C). Bars, 50 nm. (PDF) [file pbio.1001213.s009.pdf]

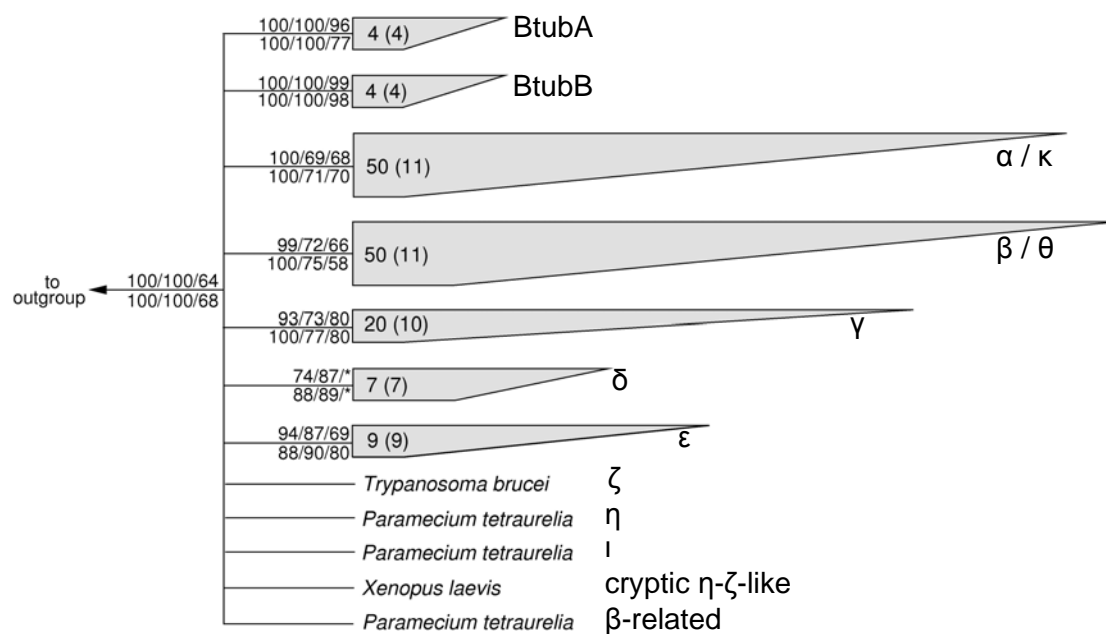

**Figure S11. Phylogenetic relationships within the Tubulin family.**

Supplement: Figure S11 — Phylogenetic relationships within the Tubulin family. Consensus tree showing only stable associations recovered in the majority of individual trees. The tubulin subfamilies α, β, γ, ε, and the groups BtubA and BtubB were recovered as monophyletic groups with relatively high support. δ-tubulins were sometimes split in two groups. With two exceptions (α-κ and β-θ), no specific associations between any of the tubulin subfamilies could be detected. Previous, less comprehensive studies made similar observations [11],[56],[57]. Likewise, BtubA and BtubB showed neither a special relationship between themselves nor an association to any tubulin subfamily and should therefore be considered as individual, novel tubulin subfamilies. Because duplication and evolution (θ and κ) of modern tubulins (β and α) are clear, the analyses do not support the hypothesis that BtubA and BtubB derived from modern α- and/or β-tubulins. The consensus is of 28 trees produced using two different alignments, seven treeing algorithms, and two different filters (Materials and Methods). Support values for six trees of the Tubulin_ClustalW database are reported at the branches, from left to right: maximum parsimony (100 bootstraps)/neighbor joining (1,000 bootstraps)/TREE-PUZZLE (1,000 puzzling steps) with a 30% (upper numbers) or 10% minimum similarity filter (lower numbers). The asterisk denotes a node, which was not recovered in the respective tree. Numbers within closed groups refer to the number of included sequences; due to calculation limits TREE-PUZZLE trees were calculated using a reduced number of sequences (number in parentheses). (PDF) [file pbio.1001213.s011.pdf]
