# Supplementary material for: Microtubules in Bacteria: Ancient Tubulins Build a Five-Protofilament Homolog of the Eukaryotic Cytoskeleton
Source: PLoS Biol. 2011 Dec 6;9(12):e1001213. doi: 10.1371/journal.pbio.1001213 (PMC3232192; doi:10.1371/journal.pbio.1001213)
Supplement: Figure S10 — Fourier transforms of simulated projections of BtubA/B tube models with different rotations. Projection images of one-start helical models of BtubA/B tubes were simulated and Fourier transformed. The spots on the subunit repeat layer line (arrowheads) were asymmetric in all cases, but the asymmetry changed depending on the rotation on the tube around its length axis (angles indicated). Since asymmetry was detected in both “B-lattice and seam” and “A-lattice without seam” tubes, the asymmetry seems to arise from the small number of protofilaments (and resulting lack of an extended “front” and ”back” side) and not from the presence of a seam. (PDF) [file pbio.1001213.s010.pdf]

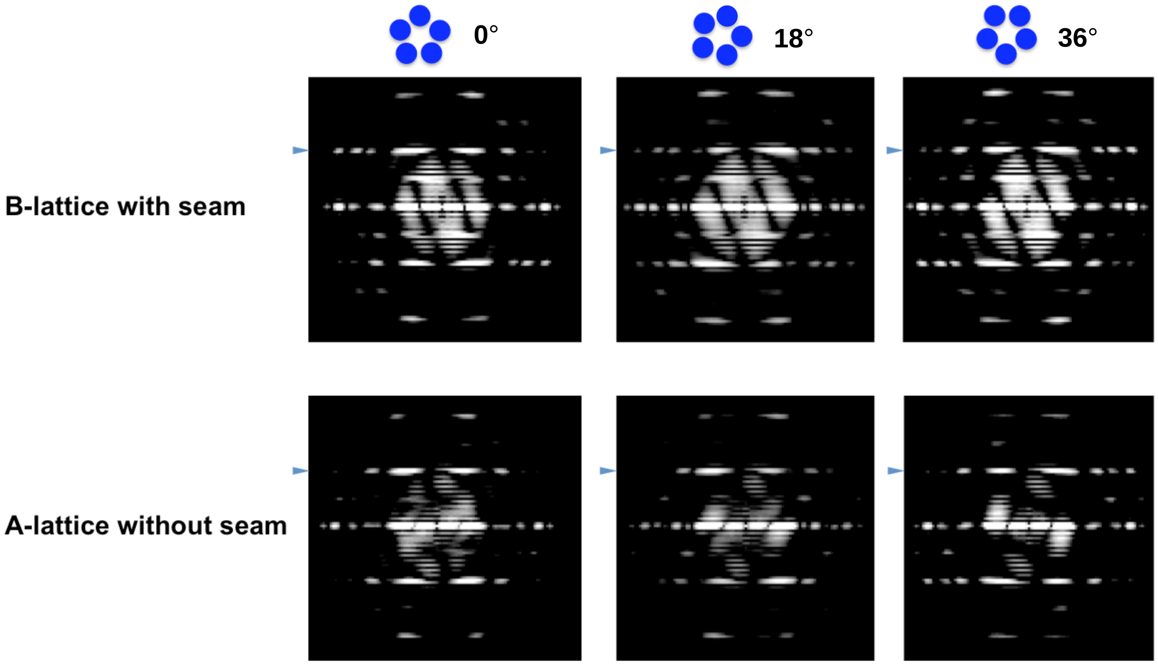

Figure S10. Fourier transforms of simulated projections of BtubA/B tube models at different rotations
