# Supplementary material for: Microtubules in Bacteria: Ancient Tubulins Build a Five-Protofilament Homolog of the Eukaryotic Cytoskeleton
Source: PLoS Biol. 2011 Dec 6;9(12):e1001213. doi: 10.1371/journal.pbio.1001213 (PMC3232192; doi:10.1371/journal.pbio.1001213)
Supplement: Table S1 — BtubA and BtubB protein motif search. PRINTS [51] was used to identify defining motifs of tubulin-related proteins (all tubulins, alpha, beta, gamma, delta, epsilon, and FtsZ) in four different BtubA and BtubB proteins (from P. dejongeii, P. vanneervenii, P. debontii operon 1, and P. debontii operon 2). The chart lists the number of motifs a protein shares with each group, as well as the “P-value” (the probability that a random sequence would achieve a higher score). BtubA and BtubB are clearly more similar to eukaryotic tubulin than to bacterial FtsZ, but they do not belong to any particular eukaryotic tubulin subfamily (as a control, the P-value of human α-tubulin with the α-tubulin subfamily is 10−121). (PDF) [file pbio.1001213.s012.pdf]

BtubA		
**Group**	**No. of motifs**	***P* value**
P. dejongeii		
Tubulin	9 of 9	$1.4 \times e^{-62}$
alpha tubulin	7 of 13	$5.9 \times e^{-17}$
beta tubulin	6 of 13	$1.4 \times e^{-14}$
epsilon tubulin	4 of 10	$1.2 \times e^{-15}$
delta tubulin	3 of 12	$1.9 \times e^{-12}$
gamma tubulin	5 of 8	$3.3 \times e^{-11}$
FtsZ	2 of 6	$8.3 \times e^{-6}$
P. vanneervenii		
Tubulin	9 of 9	$3.4 \times e^{-61}$
beta tubulin	8 of 13	$4.2 \times e^{-16}$
epsilon tubulin	4 of 10	$2.0 \times e^{-14}$
alpha tubulin	6 of 13	$6.4 \times e^{-14}$
delta tubulin	3 of 12	$1.2 \times e^{-13}$
gamma tubulin	5 of 8	$1.2 \times e^{-11}$
FtsZ	2 of 6	$1.4 \times e^{-6}$
P. debontii operon 1		
Tubulin	9 of 9	$3.5 \times e^{-61}$
epsilon tubulin	4 of 10	$2.5 \times e^{-15}$
beta tubulin	6 of 13	$6.3 \times e^{-15}$
alpha tubulin	7 of 13	$6.6 \times e^{-15}$
delta tubulin	4 of 12	$1.2 \times e^{-13}$
gamma tubulin	5 of 8	$6.5 \times e^{-11}$
FtsZ	2 of 6	$8.2 \times e^{-6}$
P. debontii operon 2		
Tubulin	9 of 9	$4.5 \times e^{-58}$
beta tubulin	7 of 13	$1.5 \times e^{-15}$
epsilon tubulin	4 of 10	$1.8 \times e^{-15}$
alpha tubulin	5 of 13	$1.4 \times e^{-14}$
gamma tubulin	4 of 8	$1.2 \times e^{-7}$
FtsZ	2 of 6	$8.3 \times e^{-6}$
delta tubulin	2 of 12	$1.5 \times e^{-5}$

BtubB		
**Group**	**No. of motifs**	***P* value**
P. dejongeii		
Tubulin	9 of 9	$1.9 \times e^{-72}$
beta tubulin	8 of 13	$4.2 \times e^{-26}$
epsilon tubulin	6 of 10	$3.3 \times e^{-19}$
alpha tubulin	5 of 13	$2.5 \times e^{-14}$
gamma tubulin	4 of 8	$1.4 \times e^{-13}$
FtsZ	3 of 6	$1.7 \times e^{-10}$
delta tubulin	2 of 12	$5.6 \times e^{-9}$
P. vanneervenii		
Tubulin	9 of 9	$1.2 \times e^{-72}$
beta tubulin	8 of 13	$3.9 \times e^{-25}$
epsilon tubulin	6 of 10	$3.4 \times e^{-20}$
gamma tubulin	5 of 8	$1.8 \times e^{-14}$
alpha tubulin	4 of 13	$2.2 \times e^{-11}$
delta tubulin	2 of 12	$5.6 \times e^{-10}$
FtsZ	2 of 6	$1.5 \times e^{-8}$
P. debontii operon 1		
Tubulin	9 of 9	$1.6 \times e^{-72}$
beta tubulin	8 of 13	$4.2 \times e^{-26}$
epsilon tubulin	6 of 10	$1.6 \times e^{-20}$
gamma tubulin	5 of 8	$1.8 \times e^{-13}$
alpha tubulin	5 of 13	$9.3 \times e^{-13}$
delta tubulin	2 of 12	$9.6 \times e^{-10}$
FtsZ	2 of 6	$4.1 \times e^{-9}$
P. debontii operon 2		
Tubulin	9 of 9	$2.2 \times e^{-73}$
beta tubulin	7 of 13	$6.3 \times e^{-24}$
epsilon tubulin	6 of 10	$2.6 \times e^{-20}$
gamma tubulin	5 of 8	$5.1 \times e^{-14}$
alpha tubulin	5 of 13	$2.3 \times e^{-12}$
FtsZ	3 of 6	$1.4 \times e^{-10}$
delta tubulin	2 of 12	$4.1 \times e^{-10}$

Table S1. BtubA and BtubB protein motif search.
