# Supplementary material for: Microtubules in Bacteria: Ancient Tubulins Build a Five-Protofilament Homolog of the Eukaryotic Cytoskeleton
Source: PLoS Biol. 2011 Dec 6;9(12):e1001213. doi: 10.1371/journal.pbio.1001213 (PMC3232192; doi:10.1371/journal.pbio.1001213)
Supplement: Table S2 — Sequence identities within the Tubulin/FtsZ superfamily. BtubA and BtubB share the highest sequence identity with eukaryotic tubulin subfamilies, but no clear relationship of BtubA or BtubB to any specific tubulin subfamily (shaded in grey) or between BtubA and BtubB could be detected. Identity values are in percentages. Pva, P. vanneervenii; Pdb, P. debontii; Pdj, P. dejongeii; Pte, Paramecium tetraurelia; Ddi, Dictyostelium discoideum; Hsa, Homo sapiens; Bth, Bacillus thuringiensis; Bce, Bacillus cereus; Hal, Halobacterium species. (PDF) [file pbio.1001213.s013.pdf]

			Tubulin															*Bacilli*		archaeal FtsZ-like			FtsZ	
			BtubA				BtubB				Alpha		Beta		Gamma	Epsilon	Delta	TubZ	RepX					
			Pdb A2	Pdb A1	Pdj	Pva	Pdb B2	Pdb B1	Pdj	Pva	Pte	Ddi	Ddi	Pte	Ddi	Hsa	Hsa	Bth	Bce	Hal3	Hal4	Hal5	Pva	Pdb
**Tubulin**	**BtubA**	Pdb A2	100																					
		Pdb A1	82	100																				
		Pdj	81	94	100																			
		Pva	81	92	93	100																		
	**BtubB**	Pdb B2	35	37	35	37	100																	
		Pdb B1	37	37	36	38	88	100																
		Pdj	35	37	34	36	90	95	100															
		Pva	36	37	36	37	84	90	90	100														
	**Alpha**	Pte	39	40	41	40	44	43	43	42	100													
		Ddi	37	37	38	39	43	42	41	42	71	100												
	**Beta**	Ddi	38	37	37	38	41	41	41	41	44	44	100											
		Pte	37	37	38	38	41	41	41	40	45	43	79	100										
	**Gamma**	Ddi	29	31	30	31	38	38	38	38	37	37	37	36	100									
	**Epsilon**	Hsa	31	30	29	29	36	35	35	34	36	34	37	37	31	100								
	**Delta**	Hsa	22	21	23	22	23	23	22	23	24	24	24	24	23	27	100							
Bacilli	**TubZ**	Bth	14	13	13	13	15	13	14	14	12	13	14	13	11	11	11	100						
	**RepX**	Bce	10	11	12	12	11	12	12	11	12	11	11	11	11	14	12	13	100					
**archaeal FtsZ-like**	**FtsZ3**	Hal	17	18	19	18	16	16	16	16	16	16	17	18	14	15	13	17	12	100				
	**FtsZ4**	Hal	17	18	19	19	20	18	19	17	17	16	17	17	17	17	15	16	12	41	100			
	**FtsZ5**	Hal	15	16	16	16	14	14	13	14	12	11	13	13	12	14	10	13	16	31	25	100		
**FtsZ**		Pva	15	16	17	17	15	14	14	15	11	13	14	14	15	15	15	11	12	19	18	17	100	
		Pdb	16	17	17	18	15	15	15	14	12	13	15	17	17	15	15	14	11	19	19	15	80	100

Table S2. Sequence identities within the Tubulin/FtsZ superfamily.
